# Hydration Asymmetry and Solvent-Mediated Charge Balancing in Microporous Carbons: An NMR Study of Aqueous and Organic Electrosorption

**DOI:** 10.1021/jacs.6c08967

**Published:** 2026-09-12

**Authors:** Ryan J. Bragg, Kieran Griffiths, Chris W. Cook, Robert A. W. Dryfe, Volker Presser, John M. Griffin

**Affiliations:** † Department of Chemistry, 4396 Lancaster University, Lancaster LA1 4YB, United Kingdom; ‡ Department of Chemistry, University of Warwick, Coventry CV4 7AL, United Kingdom; § Department of Chemistry, University of Manchester, Oxford Road, Manchester M13 9PL, United Kingdom; ∥ Henry Royce Institute, University of Manchester, Oxford Road, Manchester M13 9PL, United Kingdom; ⊥ 28391 INM - Leibniz Institute for New Materials, Campus D2 2, Saarbrücken 66123, Germany; # Department of Materials Science & Engineering, Saarland University, Campus D2 2, Saarbrücken 66123, Germany; ∇ Saarene - Saarland Center for Energy Materials and Sustainability, Campus C4 2, Saarbrücken 66123, Germany

## Abstract

Aqueous electrolytes are attracting increasing interest in supercapacitor applications due to their high sustainability and ionic conductivity relative to conventional organic systems. However, the distinct solvation properties of water and their impact on (electro)­sorption mechanisms remain poorly understood, particularly the role of hydronium (H_3_O^+^) and hydroxide (OH^–^) ions in charge storage. In this work, we utilize *in situ* and MAS NMR spectroscopy to investigate a series of aqueous and organic trifluoromethanesulfonate (triflate, OTf^–^) electrolytes within commercial activated carbon. We reveal a fundamental link between ion solvation energetics, water dissociation, and in-pore charge balancing. Our results demonstrate that an increase in the in-pore ion populations does not necessarily lead to higher capacitance; instead, improved performance is correlated with more acidic in-pore environments. Furthermore, we show that hydration asymmetry between charge-dense cations and charge-diffuse anions dictates the degree of water dissociation, providing a chemical handle to promote acidic environments and enhance capacitance. Finally, *in situ* NMR measurements elucidate the charging mechanisms of these systems, highlighting how solvent choice fundamentally alters the electrosorption process and overall electrochemical performance.

## Graphical abstract

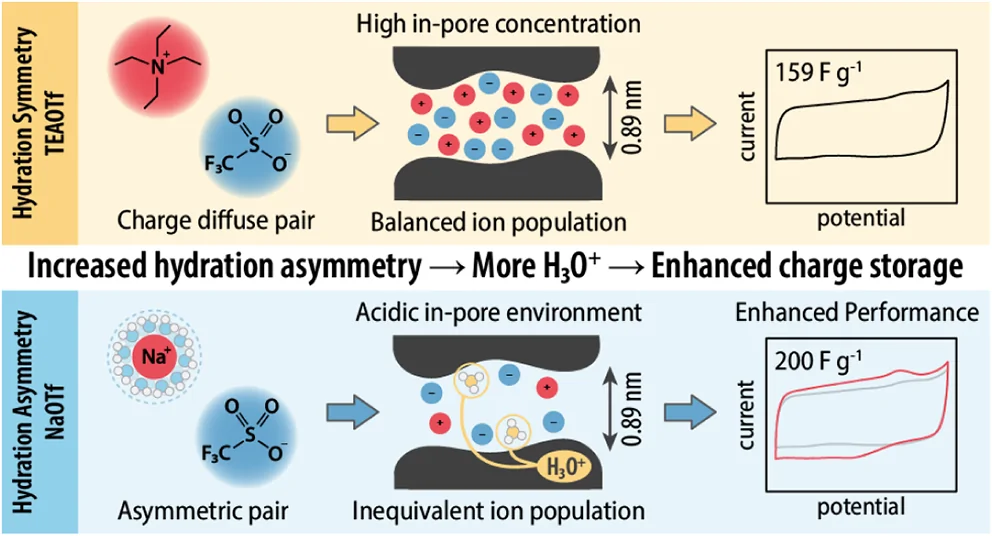

## Introduction

Electrochemical double-layer capacitors (EDLCs) are an important class of energy-storage devices, valued for their high-power outputs and long cycle lives. Charge storage in these devices relies on the formation of an electrical double layer at the electrode-electrolyte interface. This process also underpins applications such as water desalination, electrocatalysis, and electrochemical capture of carbon dioxide.^1–4^ EDLC electrodes are most commonly constructed from microporous activated carbons due to their high internal surface areas, good electrical conductivity, and ease of synthesis.

Organic electrolytes are widely used for EDLCs due to their wide voltage windows, whereas aqueous electrolytes provide higher ionic conductivity and are often more environmentally friendly.^5–7^ However, the differences in electrosorption behavior between organic and aqueous electrolytes, and the link with EDLC performance, remain poorly understood. This knowledge gap is primarily due to the complex interplay between ion solvation, micropore dimensions, and the role of solvent molecules at electrochemical interfaces.

Understanding the factors governing ion electrosorption mechanisms is challenging owing to the presence of rapid ion dynamics, disordered carbon structures, and unquantified contributions from surface functional groups. Various techniques have been employed to probe electrosorption within porous carbon electrodes, such as small-angle X-ray/neutron scattering (SAXS/SANS),^1,8,9^ attenuated total reflectance-surface-enhanced infrared absorption spectroscopy (ATR-SEIRAS),^10–12^ and electrochemical quartz crystal microbalance (EQCM).^13,14^ Additionally, nuclear magnetic resonance (NMR) spectroscopy has been widely utilized due to the well-established ring-current shielding effect in microporous carbons, which shifts the signals of adsorbed species to lower frequencies relative to bulk species, thereby enabling the study of ion adsorption mechanisms.^15–21^

Recent NMR studies have highlighted the importance of ion solvation properties in aqueous electrochemical systems. For instance, Luo et al. observed an excess population of anions relative to cations in aqueous sodium-based electrolytes within micropores, attributing this imbalance to differences in hydration enthalpies.^22^ Cervini *et al*. reinforced the impact of specific ion effects on aqueous ion populations in micropores.^23^ Expanding on this, Bragg *et al*. explored the relationship between pore size and ion-solvent interaction strength in alkali-metal-based aqueous electrolytes using *ex situ* and *in situ* NMR spectroscopy, revealing that the ion-water interaction strength influences the spontaneous partitioning of ions within microporous carbons.^24^

Unlike organic electrolytes, aqueous electrolytes can generate hydronium (H_3_O^+^) and hydroxide (OH^–^) ions, which can participate in charge balancing and storage. EQCM measurements on carbide-derived carbon electrodes with 1-ethyl-3-methyl-imidazolium (EMI^+^) bisulfate electrolyte have demonstrated that H_3_O^+^ ions participated in the charge-balancing mechanism in place of heavier EMI^+^ cations.^25^ A recent NMR study by Lyu *et al*. demonstrated that the presence of H_3_O^+^ can rationalize pH-dependent salt anion/cation population discrepancies within carbon pores.^26^ Their findings suggested that under acidic conditions, the total ion count within pores increases due to protonation of surface functional groups, which attract additional anions such as bis­(trifluoromethanesulfonyl)­imide (TFSI^–^). Comparable behavior has been observed for aqueous electrolytes within activated carbon cloths (ACCs), where larger spontaneous increases in the in-pore electrolyte concentration correlated with greater pH shifts, indicating more acidic in-pore environments.^24^ However, a predictive framework linking intrinsic ion-solvation energetics to the propensity for water dissociation and proton-mediated charge balancing remains elusive. Furthermore, a comprehensive comparison between organic and aqueous electrolytes is still lacking, particularly regarding ion-solvent interactions and their impact on electrochemical performance. Comparing these systems can help isolate the contributions of H_3_O^+^ and OH^–^ and provides a controlled baseline to evaluate how solvation-sphere energetics drive water dissociation, a process which is fundamentally absent in organic electrolytes. This interfacial dissociation is analogous to the behavior observed in bipolar membranes, where the field-induced generation of H^+^ and OH^–^ ions at the membrane junction is a central mechanism for electrodialysis and electrochemical CO_2_ capture.^27^

Here, we investigate the adsorption behavior of a series of aqueous trifluoromethanesulfonate (triflate, OTf^–^) electrolytes: LiOTf, NaOTf, and tetraethylammonium triflate (TEAOTf), which exhibit significant variations in ion-solvation interaction strengths. Beyond its role in energy storage, the triflate anion is of particular interest as it is among the most mobile per- and polyfluoroalkyl substances (PFAS); consequently, understanding its adsorption dynamics on carbon surfaces is of broader significance for environmental monitoring and remediation.^28^ Using NMR spectroscopy, we examine their behavior in both water (H_2_O) and acetonitrile (MeCN) solvents within commercial activated carbon cloth. Magic-angle-spinning (MAS) NMR experiments reveal significant in-pore anion/cation imbalances for aqueous LiOTf and NaOTf. This is rationalized by pH measurements, which show that spontaneous in-pore pH changes are governed by the hydration asymmetry between the charge-diffuse anions and charge-dense cations; electrolyte ion imbalances are subsequently offset by the formation and partitioning of H_3_O^+^ and OH^–^ across the in-pore and ex-pore environments. These results suggest that neutral bulk electrolytes do not necessarily maintain chemical neutrality within nanoconfined environments, where hydration asymmetry drives a marked deviation from bulk pH values.

In contrast, aqueous TEAOTf shows balanced TEA^+^ and OTf^–^ populations and a negligible pH effect, reinforcing the fundamental role of specific ion effects and ion-solvent interactions in determining H_3_O^+^ and OH^–^ partitioning. While interfacial charge storage is often modeled through the electrosorption of salt ions, our results reveal a distinct departure from ion stoichiometry in systems with high hydration asymmetry. We demonstrate that improved capacitance and rate performance do not correlate with increasing in-pore salt-ion occupancy;^26,29^ instead, enhanced storage is found to coincide with non-stoichiometric regimes where metal cations are largely excluded in favor of solvent-derived H_3_O^+^. The tailoring of hydration asymmetry thereby provides a molecular handle to modulate the in-pore chemical environment and optimize charge storage. This mechanism is further supported by MeCN-based electrolytes, which exhibit no in-pore anion/cation imbalance, supporting that the observed non-stoichiometry in aqueous systems stems from the formation and partitioning of H_3_O^+^ and OH^–^. Finally, *in situ* NMR measurements of LiOTf and TEAOTf electrolytes in both H_2_O and MeCN demonstrate that voltage-driven chemical shift changes are intrinsically linked to the specific ion-surface interactions facilitated by the aqueous environment, highlighting the influence of solvent choice on charging mechanisms and electrochemical performance.

## Experimental Section

### Materials

ACC-5092-20 (Kynol, referred to hereafter as ACC-20) activated carbon cloth was dried in vacuo at 60 °C for at least 12 h prior to use to remove residual water adsorbed from the atmosphere from the micropores. The average pore size (0.89 nm), which is related to the volume-weighted d_50_ value, and the internal surface area (2070 m^2^ g^–1^), derived using quenched-solid density functional theory, were determined in our previous work.^24^

Electrolytes with a molar strength of 1 M were prepared by dissolving lithium trifluoromethanesulfonate (LiOTf, Sigma-Aldrich, >99.0%), sodium trifluoromethanesulfonate (NaOTf, Sigma-Aldrich, >98.0%), and tetraethylammonium trifluoromethanesulfonate (TEAOTf, Fluorochem, >99.0%) in either deionized water or acetonitrile (MeCN, Sigma-Aldrich, >99.9%). Ionic salts were stored under an inert atmosphere following drying under vacuum (12 h). Salt masses were weighed under an argon atmosphere due to the hygroscopic nature of many reagents. Acetonitrile-based electrolytes were fully prepared under an inert atmosphere. For aqueous electrolyte samples, 15 μL of electrolyte was injected into the center of the rotor using a microsyringe in 2.5 μL increments into 3.2 mm zirconia MAS NMR rotors packed with 13 mg of ACC-20. For organic electrolyte samples, the same process was performed under an inert atmosphere. Once loaded, rotors were then sealed and left to equilibrate for 24 h.

For *in situ* experiments, circular electrodes with a diameter of 5 mm were punched from ACC-20 and soaked in the respective electrolyte for 24 h. Two-electrode *in situ* full-cells were constructed using polyether ether ketone (PEEK) cases (ePROBE GmbH) and gold mesh current collectors (Thermo Fisher Scientific, 0.064 mm diameter wires, >99.9%). Polytetrafluoroethylene spacers (2 × 0.5 mm) were placed within the cell cavity to ensure sufficient contact between components. For aqueous electrolyte systems, a polymer film separator was used (Celgard 3501). Systems using acetonitrile-based electrolytes were prepared under an inert atmosphere using borosilicate glass-fiber separators (Whatman, 0.7 μm). Cells were wrapped with polytetrafluoroethylene tape to maintain an inert environment within the cell during prolonged measurements.

### Solid-State NMR

Solid-state MAS NMR experiments were performed using a Bruker Avance III HD WB spectrometer operating at a magnetic field strength of 9.4 T (400 MHz ^1^H Larmor frequency) using a Bruker 3.2 mm HXY double-resonance probe. *In situ* solid-state NMR experiments were performed on the same spectrometer using an ePROBE static HX double-resonance NMR probe with a 10 mm coil. MAS NMR spectra were acquired at a spinning frequency of 5 kHz. Faster spinning frequencies were avoided to prevent leakage of electrolyte during spinning. ^7^Li and ^23^Na MAS NMR spectra were acquired using simple one-pulse-acquire sequences, with radiofrequency pulse durations of 4.25 μs for ^7^Li and 6 μs for ^23^Na using a pulse power of 120 and 100 W, respectively. 64 transients were acquired for each ^7^Li MAS NMR spectrum and 2048 transients for each ^23^Na MAS NMR spectrum. ^1^H MAS NMR spectra were acquired using a spin-echo experiment, with a 90° radiofrequency pulse duration of 4.5 μs pulse at 80 W and a 180° radiofrequency pulse duration of 9.0 μs pulse at 80 W. ^19^F MAS NMR spectra were acquired using a double spin-echo experiment with inversion pulses of 6.5 μs to 10.5 μs at 100 W. Recycle delays ranged from 3 to 15 s, with 64 transients being acquired for each spectrum. Recycle delays were set to >5T_1_, as determined by saturation-recovery experiments. Recycle delays ranged from 5.0 to 6.0 s for ^1^H MAS NMR experiments and ranged from 5 to 15 s for ^19^F MAS NMR experiments. Recycle delays ranged from 2.5 to 6.0 s for ^7^Li and ranged from 0.3 to 1.5 s for ^23^Na MAS NMR. ^7^Li and ^23^Na spectra were referenced using their corresponding neat 1 M electrolyte solutions at 0 ppm. ^1^H chemical shifts were referenced relative to the methyl group of alanine, set to 1.2 ppm, and ^19^F chemical shifts were set to the CF_2_ group of polytetrafluoroethylene, set to −122.7 ppm. *In situ* ^19^F NMR spectra were acquired using a double spin-echo experiment to selectively observe one electrode within the staggered cell arrangement. The *in situ* cell was oriented such that the electric field lay perpendicular to the applied field. *In situ* ^19^F NMR 90° radiofrequency pulse durations ranged from 20 μs to 30 μs using a pulse power of 140 W. Deconvolution of static spectra was performed using the DMFit software, with the Gaussian: Lorentzian ratio and peak positions allowed to freely change to give the best fit with experimental data.^30^

### pH Measurements

pH was measured using a Mettler Toledo FiveEasy FE20 pH meter with a Mettler Toledo LE438 IP67 electrode and confirmed using Cytiva PANPEHA indicator strips periodically throughout the measurement. The initial pH of a 5 mL aliquot of the aqueous electrolyte solution was measured, after which 0.5 g of ACC-20 was added to give a 10 mass% mixture. The vial containing the mixture was then sealed and stirred at 300 rpm until the pH of the solution had equilibrated. The change in H_3_O^+^ population, $\Delta n^{H_{3}O^{+}}$ , (in mmol g^–1^), was determined using

1 $$\Delta n^{H_{3}O^{+}}=\frac{\left({[H_{3}O^{+}]}_{\mathrm{initial}}-{[H_{3}O^{+}]}_{\mathrm{final}}\right)\times V}{m}$$

where [H_3_O^+^]_initial_ is the initial concentration of H_3_O^+^, [H_3_O^+^]_final_ is the equilibrated concentration of H_3_O^+^ following addition of ACC-20, *V* is the electrolyte volume (in L), and *m* is the mass of ACC-20 (in g) added to the electrolyte.

### Electrochemical Measurements

Electrochemical measurements of individual carbon electrodes were performed in a three-electrode asymmetric half-cell configuration using a custom-built polyether ether ketone body equipped with spring-loaded Ti pistons.^31^ Disc-shaped working electrodes (∼3 mg) and larger counter electrodes (∼10 mg) were punched from dried ACC-20 (under vacuum, 60 °C, 12 h) and soaked together in 5 mL of electrolyte for 12 h. Half cells comprising aqueous electrolytes were assembled using a BASi Ag/AgCl (3 M NaCl) reference electrode with a formal reduction potential of +0.209 V (*vs*. NHE) and Celgard 3501 porous membrane as the separator. For organic systems, half-cells were fabricated under argon inside a glovebox. A platinum wire was used as a quasi-reference electrode, and a borosilicate glass fiber mat was used as the separator. Cyclic voltammetry (CV) was performed at 1 mV s^–1^, 5 mV s^–1^, 10 mV s^–1^, 50 mV s^–1^, 100 mV s^–1^, and 200 mV s^–1^ to determine rate performance. Capacitances, *C* (in F), were determined from cyclic voltammograms using

2 $$C=\frac{\int_{V_{1}}^{V_{2}}IdV}{\frac{dV}{dt}\times \left|V_{2}-V_{1}\right|}$$

where *I* is the discharge current (in A), |*V*_2_ – *V*_1_| is the voltage window, *V*_1_ and *V*_2_ are the bounds of the discharge voltage window in which capacitive behavior is observed (in V), and d*V*/d*t* is the scan rate (in V s^–1^). Capacitances were determined from the tenth cycle. For half-cell measurements, the specific capacitance, *C*_s_ (in F g^–1^), was extracted by dividing the capacitance by the mass of the working electrode. For two-electrode full-cell measurements, the mass of the working and counter electrodes was assumed equivalent, with *C*_s_ being defined by

3 $$C_{s}=\frac{2C_{T}}{m_{\mathrm{avg.}}}$$

where *C*_T_ is the cell capacitance and *m*_avg._ is the average mass of a single electrode, given by *m*_avg._ = (*m*_e1_ *+ m*_e2_)/2.^32^

## Results

### Spontaneous Adsorption of Triflate Electrolytes

MAS NMR measurements were performed on ACC-20 soaked with 1 M solutions of triflate (OTf^–^) salts of Na^+^, Li^+^, and tetraethylammonium (TEA^+^) in either deionized water or MeCN. ^23^Na and ^7^Li measurements probe metal cations, ^1^H measurements probe H_2_O and TEA^+^ cations, and ^19^F measurements probe OTf^–^ anions. In-pore species are shifted toward more negative chemical shifts by several ppm due to ring-current shielding induced by the nearby aromatic carbon surfaces.^17,24^ Figure 1a shows representative ^1^H, ^7^Li, and ^19^F MAS NMR spectra of ACC-20 (13 mg) loaded with 1 M aqueous LiOTf (15 μL), in which the in-pore and ex-pore resonances for H_2_O, Li^+^, and OTf^–^ species are highlighted. From these spectra, the measured in-pore and ex-pore resonances for the ions and solvent species are then integrated to quantify the local in-pore and ex-pore ionic populations (Figure 1b).^17–19,33,34^ For narrative readability throughout this work, we refer to these quantified populations in terms of a simple operational term “concentration.” Rigorously, because NMR signal integration yields nuclear spin populations rather than volume-derived molarities, these values represent normalized local mole ratio, *χ*_norm_, as described in our previous work.^24^ Specifically, the local concentration in each environment is determined by calculating the ratio of electrolyte ions to solvent molecules (*n*_ion_/*n*_solvent_) and normalizing it against the corresponding ion-to-solvent mole ratio of the neat, unconfined stock solution. Equation 4 demonstrates this approach for the in-pore environment:

4 $$\chi_{\mathrm{in‐pore},\;\mathrm{norm}}=\frac{\left(n_{\mathrm{in‐pore},\;\mathrm{ion}}/n_{\mathrm{in‐pore},\;\mathrm{solvent}}\right)}{\left(n_{\mathrm{neat},\;\mathrm{ion}}/n_{\mathrm{neat},\;\mathrm{solvent}}\right)}$$

**Figure 1:**
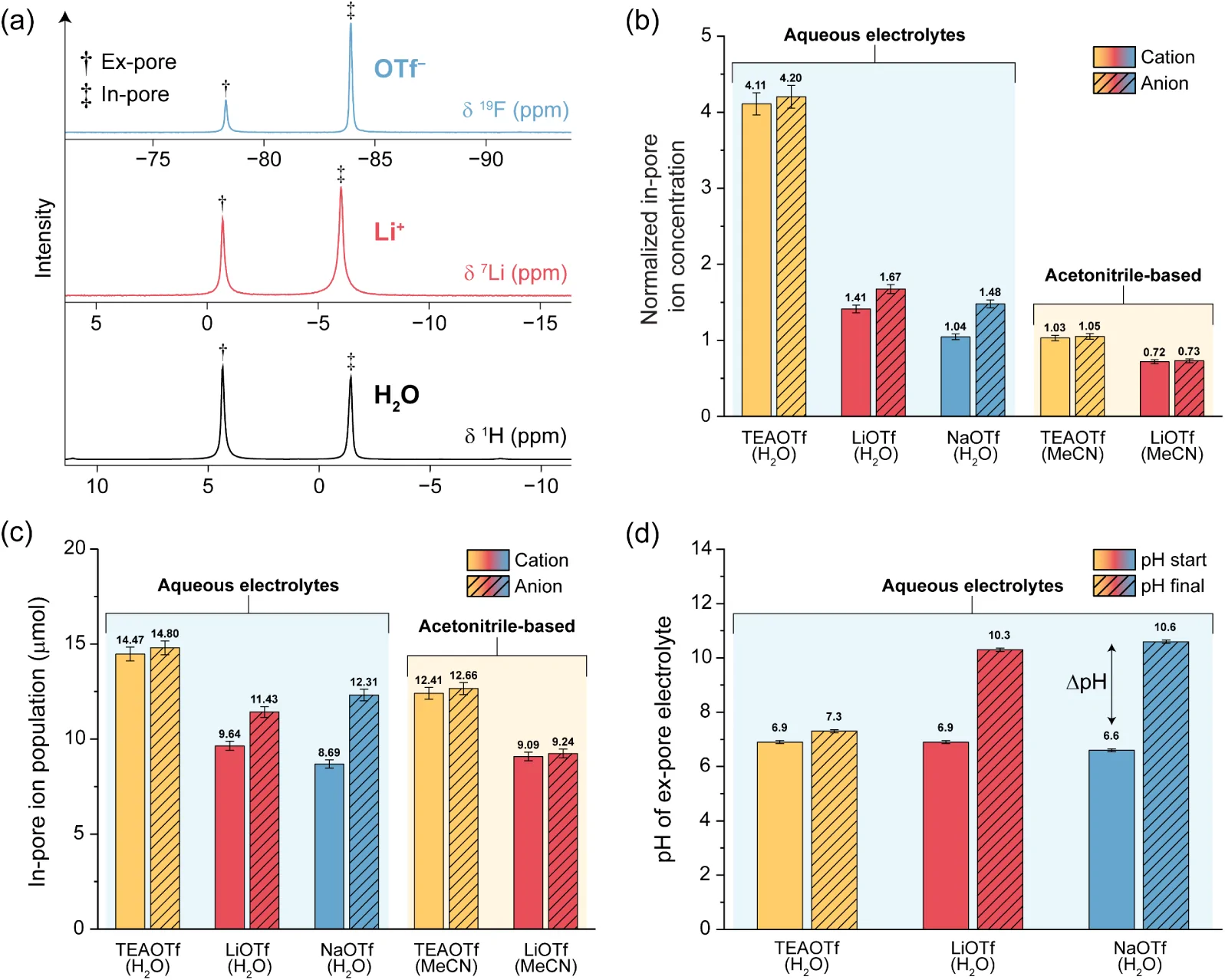
(a) ^1^H, ^7^Li, and ^19^F MAS NMR spectra for ACC-20 (13 mg) loaded with 1 M aqueous LiOTf (15 μL). (b) In-pore ion concentrations of triflate electrolytes normalized by the ionic concentrations of their 1 M neat solutions. (c) Absolute in-pore ion populations derived from deconvolution of MAS NMR spectra (15 μL of electrolyte in 13 mg of ACC-20). Error bars for concentrations and populations represent the root-mean square of 3 independent fits to the experimental data, combined with a volumetric measurement uncertainty of 1.5%. (d) pH of electrolyte solution before and after the addition of 10 wt % ACC-20. Error bars represent an experimental uncertainty of ±0.06 pH derived from replicate measurements.

By treating the co-confined solvent molecules as an internal volume proxy, and assuming the molar volume of the solvent remains roughly consistent upon confinement, this normalization ensures that a reported normalized concentration value greater than (or less than 1) directly corresponds to local ionic enrichment (or depletion) relative to the bulk electrolyte.

Across the aqueous triflate series, normalized in-pore ion concentrations are higher than 1, showing that electrolyte ions preferentially occupy the in-pore environment (Figure 1b). This aligns with behavior previously seen for TFSI^–^ and ClO_4_^–^ based electrolytes.^22,26^ Indeed, OTf^–^, TFSI^–^, and ClO_4_^–^ are all considered to be hydrophobic ions with ion-H_2_O interaction energies that are smaller than the H_2_O–H_2_O interaction energy, and negative transfer free energies from H_2_O to less polar solvents.^35^ These properties provide a strong propensity for aqueous OTf^–^ salts to spontaneously enter micropores because the anion hydration sphere can be easily distorted or partially removed, and interaction energies between the anion and the predominantly nonpolar carbon surface can offset or exceed anion-H_2_O interaction energies.^24,36^ TEAOTf displays a significantly higher in-pore ion concentration than LiOTf and NaOTf. Indeed, TEA^+^ has a significantly lower hydration enthalpy than Li^+^ and Na^+^,^37^ which facilitates pore entry of TEA^+^ and enables TEAOTf to adsorb such that there is a higher population inside the pores. This is supported by a comparison between TEACl and NaCl, where TEA^+^ shows significantly higher uptake than Na^+^ even in the presence of the strongly solvated Cl^–^ anion (Supporting Information, Figure S1).^22–24^ In contrast, Li^+^ and Na^+^ are strongly hydrated cations that incur an energy penalty upon pore entry due to the distortion of the hydration sphere.^38,39^ As a result, Na^+^ and Li^+^ ions preferentially occupy the ex-pore environment, and electrostatic anion–cation interactions limit OTf^–^ insertion into micropores.

This disparity in cation solvation energies results in a significant deviation from stoichiometric adsorption for the LiOTf and NaOTf systems. While the weakly hydrated OTf^–^ anions readily occupy the pore space, the high desolvation penalty of the charge-dense Li^+^ and Na^+^ cations leads to their relative exclusion. Such a population imbalance, which is notably absent in the hydration-symmetric TEAOTf system, highlights hydration asymmetry as a primary governing factor in spontaneous adsorption. The resulting local charge disparity suggests that the energetic drive for anion adsorption can partially overcome the electrostatic requirement for cation-anion pairing, necessitating the involvement of alternative solvent-derived species to maintain pore neutrality. Crucially, as shown by the absolute population metrics (Figure 1c), charge balancing between electrolyte salt anions and cations remains the dominant mode of charge balancing for much of the in-pore population (*e.g.*, 8.69 μmol of Na^+^ is adsorbed with 12.31 μmol of OTf^–^, therefore 70% of OTf^–^ are balanced by Na^+^).

It is important to consider the physical state of these constituent ions within the micropores. Under strict nanoconfinement, the relative permittivity (dielectric constant) of water is known to be severely reduced compared to its bulk value, with significant dielectric suppression reported in sub-nanometer channels and capillaries.^40–42^ A similar reduction in static permittivity is expected for confined acetonitrile.^43^ This suppression of dielectric screening inevitably enhances the thermodynamic favorability of partial salt association and ion-pairing inside the pores. However, because MAS NMR inherently integrates the total nuclear spin population, the macroscopic stoichiometric imbalances observed for aqueous NaOTf and LiOTf (Figure 1c) are absolute. Thus, while enhanced ion-pairing may govern the local structural arrangement of the cations that do successfully enter the pore, it cannot account for the observed surplus of in-pore OTf^–^ relative to the cations. This absolute stoichiometric imbalance confirms that the observed anion excess represents a genuine, localized net charge that necessitates the active involvement of alternative, solvent-derived ionic species to maintain electroneutrality. The involvement of solvent-derived species is not driven by a higher intrinsic affinity over the salt pairs, but is instead a localized, compensatory response forced by inequivalent salt ion populations.

For MeCN-based electrolytes, the in-pore anion and cation populations are equal for both LiOTf and TEAOTf to within experimental error (NaOTf was not studied due to low solubility in MeCN) (Figure 1c). This balanced distribution stands in stark contrast to the aqueous systems, where population imbalances are observed.^22,26^ The stoichiometric nature of ion uptake in MeCN implies that the charge-compensation mechanism in organic systems is strictly coupled to the salt ions themselves. This strict stoichiometry is a direct consequence of the dielectric properties of the organic solvent. Because acetonitrile possesses a lower bulk relative permittivity than water (a property that is further suppressed under nanoconfinement), ion-pairing is heavily favored. This low-dielectric environment effectively binds the cations and anions into strongly correlated pairs, thermodynamically compelling them to enter and occupy the pores in equal 1:1 ratios. This underscores a fundamental difference between aqueous and organic systems. While salt ions are self-balancing in MeCN, the breakdown of stoichiometry in aqueous electrolytes suggests that charge neutrality must be maintained through the active involvement of alternative ionic species. Additionally, MeCN-based electrolytes exhibit significantly lower total in-pore ion concentrations compared to their aqueous counterparts, with LiOTf ions preferentially residing outside the pores. The origin of this reduced adsorbed population likely arises from the interplay between solvation free energies (Δ*G*_s_) and solvated radii. While Δ*G*_s_ for Li^+^ is comparable in both H_2_O and MeCN,^35,44,45^ the solvation properties of OTf^–^ in MeCN, which, to our knowledge, remain unreported in the literature, may provide a higher energetic barrier to entry, leading to reduced uptake into pores. Furthermore, the higher in-pore population of TEAOTf compared to LiOTf in MeCN indicates that the barrier for TEA^+^ insertion remains lower than that of the more tightly solvated Li^+^ ion in the organic phase. Ultimately, the strict stoichiometry of the organic systems confirms that the population imbalances seen in water are unique to the aqueous environment, pointing toward the recruitment of solvent-derived species to resolve charge disparity.

### The Role of H_3_O^+^ and OH^–^ Ions

To gain insight into the origin of the in-pore anion/cation imbalances in aqueous electrolytes, pH measurements were carried out. Upon the addition of 10 wt % ACC-20 to 5 mL of aqueous NaOTf, the ex-pore solution showed a pH change, ΔpH_ex‑pore_, of +4.0 ± 0.06 (Figure 1d), corresponding to the presence of an excess of OH^–^ in the ex-pore environment. pH changes toward increased basicity are often observed upon the addition of aqueous solutions to activated carbons. They are commonly ascribed to the presence of incombustible metal impurities (ash) retained from organic precursors used in synthesis. However, ACC-20 is derived from a polymer precursor that does not contain metals; indeed, this is confirmed by elemental analysis, which shows a negligible ash content (<1.5%) (Supporting Information, Table S1).^46^ Furthermore, a negligible ΔpH_ex‑pore_ was observed upon the addition of ACC-20 to deionized water, which shows that the pH change is directly related to the presence and properties of the electrolyte ions. A similar, albeit smaller increase of ΔpH_ex‑pore_ = +3.4 ± 0.06 was also observed for LiOTf.

In contrast, TEAOTf exhibited a small ΔpH_ex‑pore_ of +0.4 ± 0.06. Aqueous TEAOTf also exhibits the smallest discrepancy between the in-pore anion and cation populations (Figure 1c). This indicates that the in-pore anion/cation imbalance observed by NMR is related to ΔpH_ex‑pore_, with solvent-derived H_3_O^+^ and OH^–^ actively partaking in balancing ionic charge across the system. We note that MAS NMR in-pore/ex-pore population ratios for weakly hydrated ions such as OTf^–^ show a dependence on the total loading volume,^24^ and the conditions used here (15 μL of electrolyte with 13 mg of activated carbon) differ from the pH measurements where the electrolyte was in excess (5 mL with 0.5 g). For this reason, we would not expect the precise anion/cation population imbalance to match the H_3_O^+^ population inferred from the pH measurements. However, when normalized relative to NaOTf, the trend in the magnitude of the in-pore ion imbalance across the electrolyte series follows the same trend as the change in the H_3_O^+^ population determined using eq 1 (Supporting Information, Figure S2).

The link between anion/cation population imbalances and ΔpH_ex‑pore_ is further supported by comparison with MeCN-based electrolytes, which both exhibit negligible in-pore anion/cation imbalances (Figure 1c). This shows that for non-aqueous electrolytes where H_3_O^+^ and OH^–^ are absent, the electrolyte ions are forced to occupy the in-pore environment with equal populations to maintain local charge neutrality. In contrast, for aqueous electrolytes exhibiting in-pore anion/cation imbalances, local charge neutrality is maintained through water dissociation associated with the asymmetric adsorption of salt ions. In the systems studied here, the net negative charge from surplus OTf^–^ within the pores is balanced by the generated H_3_O^+^, and the net positive charge from excess cations (Na^+^, Li^+^, or to a much lesser extent TEA^+^) outside the pores is balanced by the corresponding OH^–^. This confinement-induced water dissociation and subsequent partitioning of the H_3_O^+^ and OH^–^ products allows the system to minimize overall free energy by desolvating charge-diffuse anions inside the pores while leaving an excess of charge-dense metal cations outside the pores, without violating local or global electroneutrality.

### Electrochemical Analysis of Aqueous Triflate Electrolytes

The impact of aqueous cation properties on capacitive energy storage was studied using half-cells containing aqueous triflate electrolytes and ACC-20 electrodes. For full potential sweeps from +0.8 V to −0.8 V vs. Ag/AgCl (3 M NaCl) (Figure 2a), aqueous NaOTf exhibits the largest specific capacitance, *C*_s_ (200 ± 6 F g^–1^), followed by LiOTf (180 ± 5 F g^–1^) and TEAOTf (159 ± 5 F g^–1^). This trend is maintained across the negative voltage window (Figure 2b–d), with specific capacitances of 174 ± 5 F g^–1^ (NaOTf), 148 ± 5 F g^–1^ (LiOTf), and 138 ± 5 F g^–1^ (TEAOTf). This finding is notably surprising when compared to our NMR results: NaOTf exhibits the lowest spontaneous in-pore ion concentration yet yields the highest energy storage capacity. This demonstrates that interfacial charge storage in these systems is not a simple function of in-pore electrolyte ion concentration but is instead governed by a more complex interplay of ionic species.

**Figure 2:**
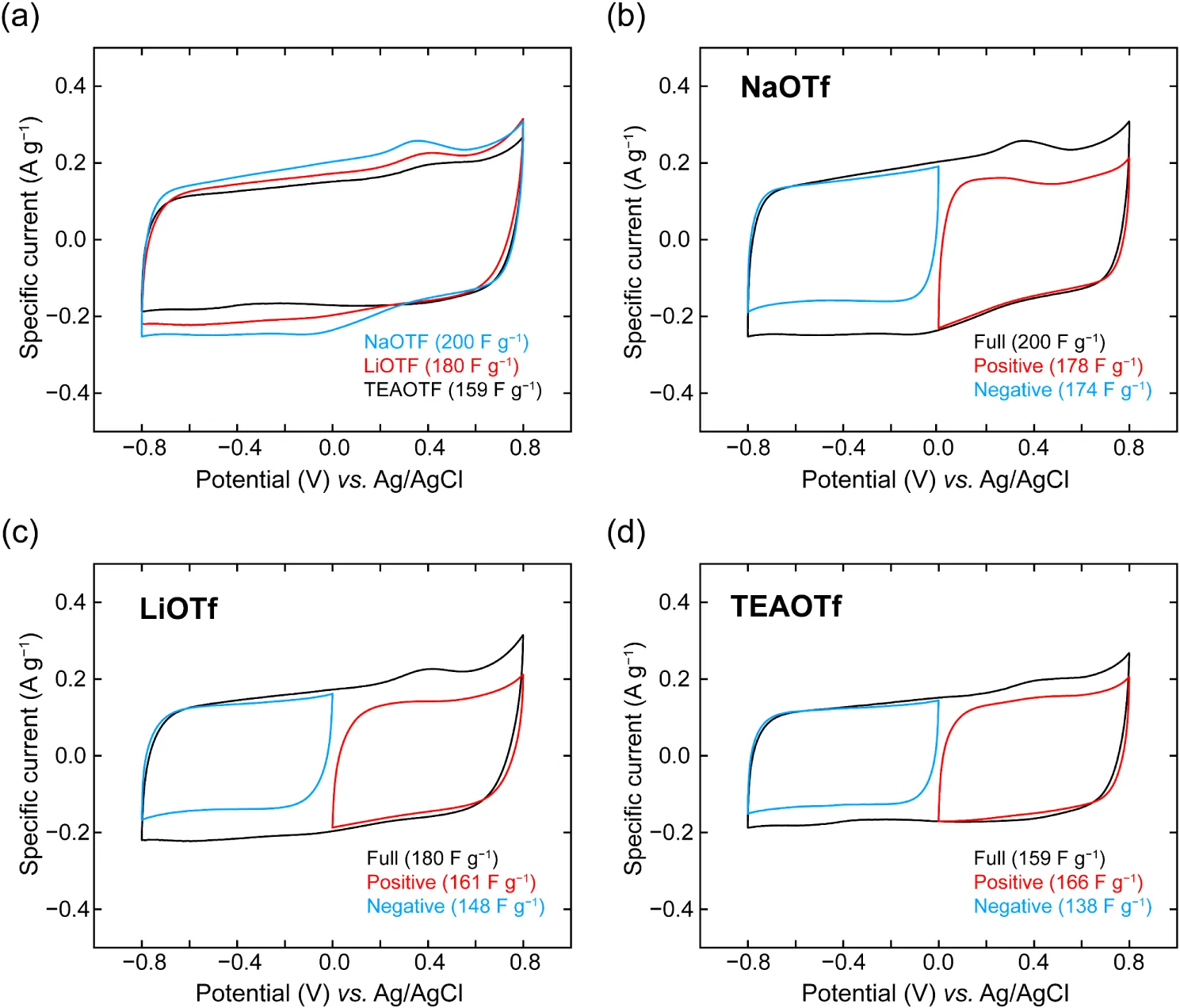
(a) Three-electrode cyclic voltammograms of aqueous 1 M LiOTf, NaOTf, and TEAOTf electrolytes cycled between ±0.8 V vs. Ag/AgCl reference electrode (+0.209 V vs. NHE). Superimposed three-electrode cyclic voltammograms cycled from 0 V to +0.8 V, 0 V to −0.8 V, and ±0.8 V are shown for cells comprising (b) NaOTf, (c) LiOTf, and (d) TEAOTf. A scan rate of 1 mV s^–1^ was used, and the 10th cycle is displayed in each instance.

In cyclic voltammograms across the full potential window (Figure 2a), a broad peak is observed at approximately +0.35 V *vs*. Ag/AgCl (3 M NaCl) during charging for all three aqueous electrolytes. The magnitude of this current peak scales directly with the acidity of the in-pore environment, being largest for NaOTf (most acidic) and smallest for TEAOTf (least acidic), as inferred from pH measurements (Figure 1d). Similar cyclic voltammogram shapes have been previously correlated with (i) redox interactions with surface functional groups,^47–50^ (ii) gradual potential-driven rearrangement of ionic species in the near-surface region,^51^ and (iii) desolvation of ionic species upon insertion into micropores, creating image charges in the carbon electrodes and facilitating a more efficient screening of ionic charges.^52,53^ Increasing the positive potential beyond +0.65 V *vs.* Ag/AgCl (3 M NaCl), up to the limit of +0.80 V, results in a further increase in current, indicating the onset of electrolyte breakdown.^54^

Recent work by Lyu *et al*. on aqueous acidic and alkali LiTFSI solutions within activated carbon demonstrated that acidic electrolytes exhibited higher in-pore ion concentrations and increased capacitances.^26^ While they attributed this to a higher number of ions supporting greater charge storage, our results reveal that high capacitance can be achieved in a distinctly different regime. In this study, the largest *C*_s_ is observed for the electrolyte with the lowest in-pore ion concentrations (NaOTf), and *C*_s_ reduces with increasing in-pore ion concentration (NaOTf > LiOTf > TEAOTf). This finding is a key takeaway of our study: interfacial charge storage efficiency is not a simple function of in-pore salt concentration. Instead, our results suggest that specific capacitance correlates with the H_3_O^+^ concentration within the in-pore environment, a state that arises as a direct consequence of the hydration asymmetry inherent to the electrolyte. This is supported by an analysis of *C*_s_ normalized per adsorbed ion (Supporting Information, Figure S3), which follows a positive trend with pH change. This suggests that the chemical environment influences storage efficiency independently of total ion density, potentially due to the increased propensity for reversible Faradaic reactions between H_3_O^+^ ions and surface functional groups.

In terms of rate performance (Supporting Information, Figure S4a), NaOTf outperforms the series, maintaining the highest *C*_*s*_ across all scan rates despite having the lowest in-pore concentration. This is attributed to the synergistic effect of reduced interfacial congestion due to the lower spontaneous in-pore ion concentration,^24,53^ and the involvement of high mobility solvent-derived charge carriers (H_3_O^+^).^55^ Evaluations of capacitive kinetics in the literature demonstrate that scan-rate dependence in porous carbons is governed by ohmic potential drops and can be modeled following transmission line theory.^56,57^ However, the capacitance of the aqueous systems herein exhibited a broad, quasi-linear decay with respect to logarithmic scan rate, precluding the extraction of bounded resistance parameters using standard sigmoidal fits predicted by transmission line models. Consequently, the rate performance is evaluated empirically as a measure of capacity retention. A reduced capacitance fade between 0.5 mV s^–1^ and 200 mV s^–1^ was also observed for NaOTf relative to LiOTf and TEAOTf (capacitance loss of 82% vs 91% and 93%, respectively).

The three-electrode CV data reveal significant asymmetry in the specific capacitance, Δ*C* (Δ*C* = *C* ^+^ – *C* ^+^, where *C* ^+^ and *C* ^–^ are the *C*_s_ values from the positive and negative cyclic voltammograms, respectively). Δ*C* correlates with the spontaneous adsorption properties (Supporting Information, Figure S4c), with TEAOTf giving the largest Δ*C* (28 ± 7 F g^–1^), followed by LiOTf (13 ± 7 F g^–1^) and then NaOTf (−4 ± 8 F g^–1^). Together, these results show that aqueous electrolytes with larger anion/cation population imbalances (Figure 1c) and larger ΔpH_ex‑pore_ (Figure 1d) under spontaneous adsorption conditions exhibit larger *C*_*s*_ and smaller Δ*C* values, in addition to improved rate performance.

To isolate the role of water, half-cell cyclic voltammograms were recorded with 1 M LiOTf and TEAOTf in MeCN (Figure 3a). Here, the capacitance trend is reversed compared to the aqueous systems; TEAOTf exhibits a larger *C*_s_ (145 ± 4 F g^–1^) than LiOTf (114 ± 3 F g^–1^). This reversal highlights that in organic media, where charge neutrality is strictly coupled to the salt ions, capacitance is dictated by the kinetic availability of ions within the micropores. For TEAOTf, the higher spontaneous occupancy provides a local reservoir of ions that can rapidly redistribute upon potential change. Conversely, the lower in-pore population of LiOTf necessitates the diffusion of solvated ions from the bulk through the tortuous micropore network, resulting in a more resistive charging profile (Figure 3b).^58^ For both electrolytes, the increase in current at approximately ±1.2 V (*vs*. Pt) indicates the onset of electrochemical breakdown.

**Figure 3:**
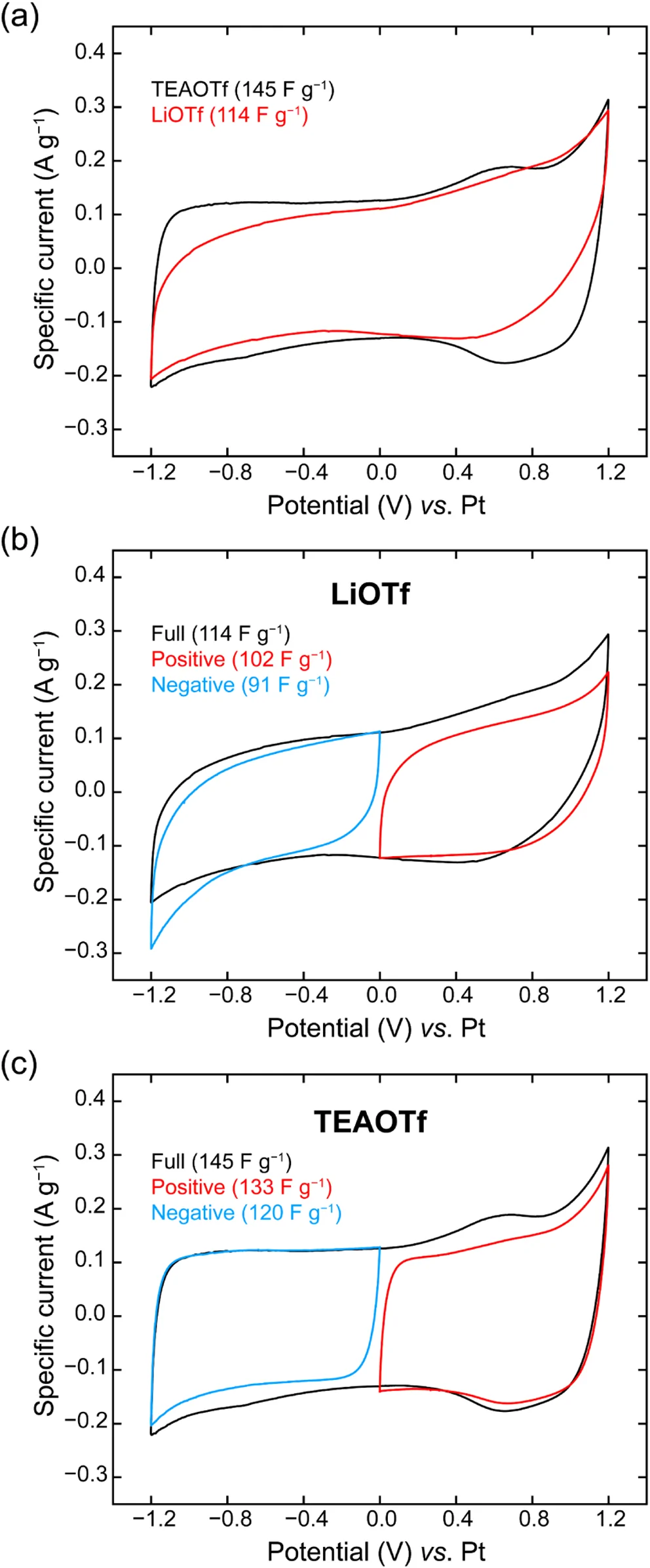
(a) Overlaid half-cell cyclic voltammograms of 1 M acetonitrile-based LiOTf and TEAOTf electrolytes cycled between ±1.2 V vs. Pt. Superimposed three-electrode cyclic voltammograms cycled from 0 V to +1.2 V, 0 V to −1.2 V, and ±1.2 V to are shown for cells comprising (b) LiOTf and (c) TEAOTf. A scan rate of 1 mV s^–1^ was applied, and the 10th cycle is displayed in each instance.

The cyclic voltammogram of the TEAOTf cell under a full potential sweep reveals distinct current peaks at +0.5 V (*vs*. Pt) during charging and +0.6 V during discharging. These peaks are diminished in the positive-only cycle (Figure 3c). Since the MeCN-based TEAOTf system lacks OH^–^ and H_3_O^+^, this behavior is attributed to a gradual, potential-driven rearrangement of OTf^–^ anions at the carbon surface.^51^ Starting from a negative potential, the near-surface region is primarily occupied by cations; as the potential becomes more positive, anion infiltration occurs gradually (corroborated by *in situ* NMR, *vide infra*). This results in a continuous and linear transition from a cation-rich to an anion-dominated interface in place of a sharp inversion in local charge, giving rise to a gradual current increase and broad peaks across the positive charge and discharge. These features are not observed for MeCN-based LiOTf, possibly linked to the increased resistance of the cell and the slower capacitive response of the solvated Li^+^ ions (Figure 3b).

### *In Situ* NMR

To gain direct insight into the charging mechanisms of the triflate electrolytes, *in situ* NMR experiments were carried out on supercapacitor cells comprising symmetrical ACC-20 electrodes in “staggered” geometry so that a single electrode is observed.^24,33^ ^19^F NMR spectra of cells containing aqueous LiOTf, NaOTf, and TEAOTf are shown in Figure 4. In each spectrum, an intense ex-pore resonance is observed, together with sharp features due to magnetic susceptibility effects from cell components.^16^ The in-pore resonance is shifted by 4–6 ppm to a more negative chemical shift.^17^

**Figure 4:**
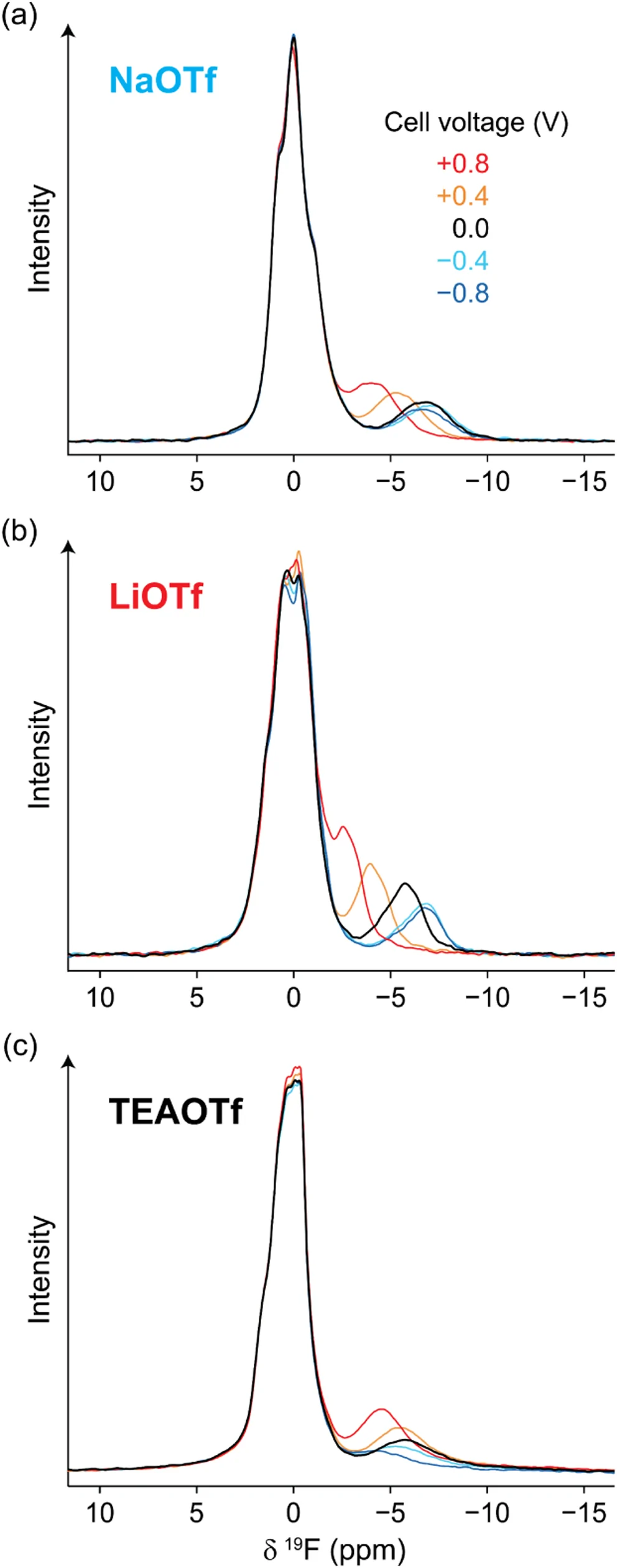
*In situ* ^19^F NMR spectra of supercapacitor cells comprising 1 M aqueous solutions of (a) NaOTf, (b) LiOTf, and (c) TEAOTf, acquired at different held cell voltages. Spectra are referenced such that the center of the ex-pore triflate resonance is at 0 ppm.

For each cell, applying positive cell voltages up to +0.8 V causes the in-pore OTf^–^ anion population to increase, consistent with a counterion adsorption charging mechanism. However, the magnitude of this response varies significantly across the electrolytes: Notably, TEAOTf shows the largest increase (+0.64 ± 0.05), compared to LiOTf (+0.45 ± 0.05) and NaOTf (+0.43 ± 0.05) (Figure 5a). Conversely, under negative cell voltages, the OTf^–^ population decreases, signifying co-ion expulsion.^34^ The most pronounced decrease in the in-pore anion population was again observed for TEAOTf (−0.52 ± 0.02), followed by LiOTf (−0.42 ± 0.02) and NaOTf (−0.19 ± 0.03). This shows that in-pore OTf^–^ population responds most strongly to the applied cell voltage in TEAOTf, with a reduced response being observed in NaOTf. Since the reduced response of the OTf^–^ population for NaOTf does not correlate with a reduced stored charge across the cell (Supporting Information, Figure S5), the relative inactivity of the triflate anion implies that other charged species (*e.g*., Na^+^, H_3_O^+^, and OH^–^) contribute to charge compensation at the electrode surface. This correlates with the increased concentration of in-pore H_3_O^+^ and ex-pore OH^–^ for NaOTf compared to TEAOTf, as quantified by the pH measurements and inferred from the NMR-derived cation/anion population imbalances. To assess whether this electrosorption deficit might be compensated by the counter-cations, complementary *in situ* ^7^Li and ^23^Na NMR was performed on separate cells containing the aqueous LiOTf and NaOTf systems (Supporting Information, Figures S6, S7). The *in-pore* Li^+^ and Na^+^ populations only show small changes over the potential range studied, suggesting limited participation of these species in charge balancing.

**Figure 5:**
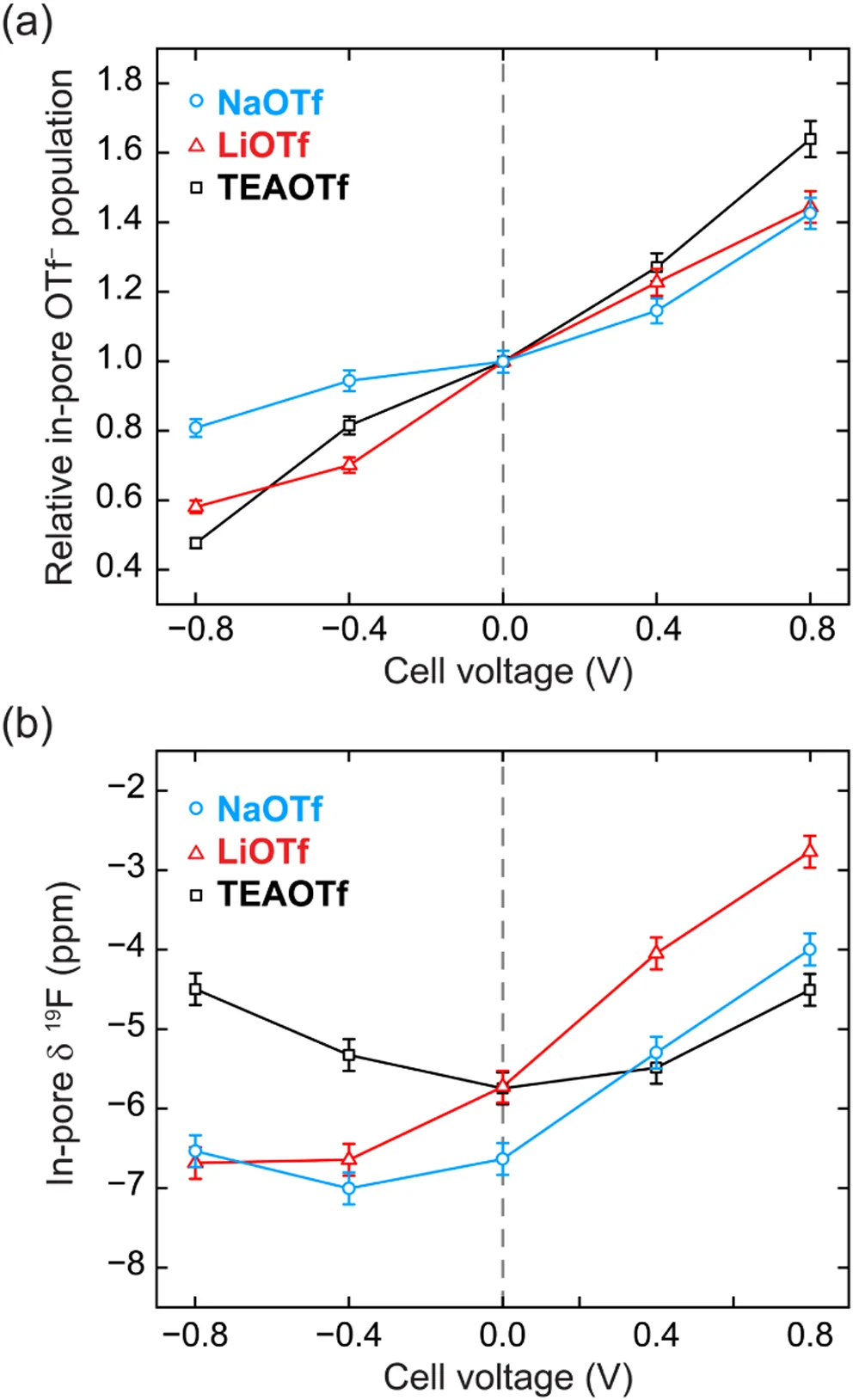
(a) Voltage-dependent responses of in-pore triflate anion populations for *in situ* cells comprising 1 M aqueous solutions of NaOTf, LiOTf, and TEAOTf. Error bars represent an ensemble relative standard deviation (±3.15%) derived from 3 independent fits of spectra under varying phasing and line-broadening configurations. (b) In-pore ^19^F chemical shift as a function of cell voltage for devices comprising 1 M aqueous NaOTf, LiOTf, and TEAOTf. Chemical shift errors (±0.2 ppm) are determined from the full width half maximum (FWHM) of the average in-pore resonance divided by the signal-to-noise ratio.

Chemical shift variations of the in-pore ^19^F resonances were also analyzed (Figure 5b). The in-pore resonance for the aqueous TEAOTf shifts toward a less negative chemical shift for both positive and negative electrode polarities. This behavior has been previously reported for organic electrolytes and ionic liquids and has been attributed to the formation of unpaired electrons or holes at the carbon surface during charging, which results in increased paramagnetic contributions to the in-pore chemical shift.^17,34,59^

For LiOTf and NaOTf, the in-pore resonances also shift to a more positive chemical shift under positive potentials. However, they exhibit an unusual diamagnetic shift to more negative chemical shifts under negative potential (Figure 5b). While large diamagnetic shifts (∼20 ppm) have been attributed to ion desolvation in extremely narrow micropores,^60^ the shifts here are notably smaller (∼2 ppm) and nucleus-independent (Supporting Information, Figure S6). Because this asymmetric shift is absent in TEAOTf (the only system lacking a significant ΔpH_ex‑pore_), we attribute this behavior to potential-driven interactions between H_3_O^+^ and the carbon surface. These interactions likely alter the local electronic structure of the anode or induce a shift in the potential of zero charge (PZC), effectively rescaling the voltage response of the observed ions. This is supported by the response of the in-pore ^19^F resonance for NaOTf, where the in-pore resonance began to shift in a positive direction, returning to a symmetric response at a cell voltage of −0.4 V (Figure 5b).

To explore the influence of the solvent on the charging mechanism, *in situ* NMR experiments were performed on cells containing MeCN-based electrolytes (Figure 6a,b). In-pore OTf^–^ population is compared with that for aqueous electrolytes in Figure 7a,b. Under negative electrode polarization, LiOTf in MeCN exhibits a decrease in the in-pore OTf^–^ concentration that is comparable to the aqueous electrolyte and consistent with a co-ion expulsion mechanism (Figure 7a).

**Figure 6:**
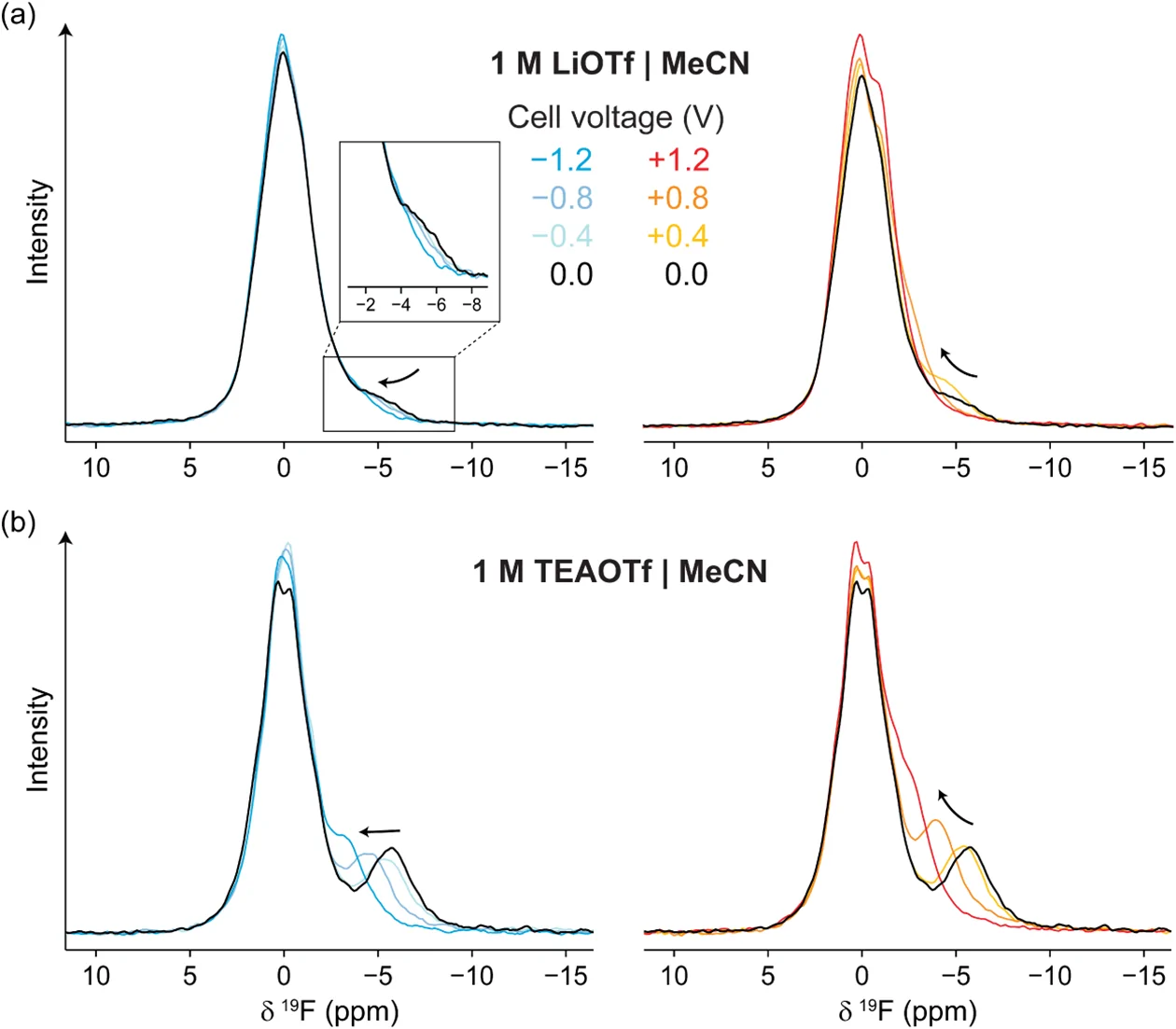
*In situ* ^19^F NMR spectra of supercapacitor cells comprising 1 M MeCN-based solutions of (a) LiOTf and (b) TEAOTf, acquired at different held cell voltages. Spectra are referenced such that the center of the ex-pore triflate resonance is at 0 ppm. Black arrows are drawn to highlight the voltage-driven movement of the in-pore resonance.

**Figure 7:**
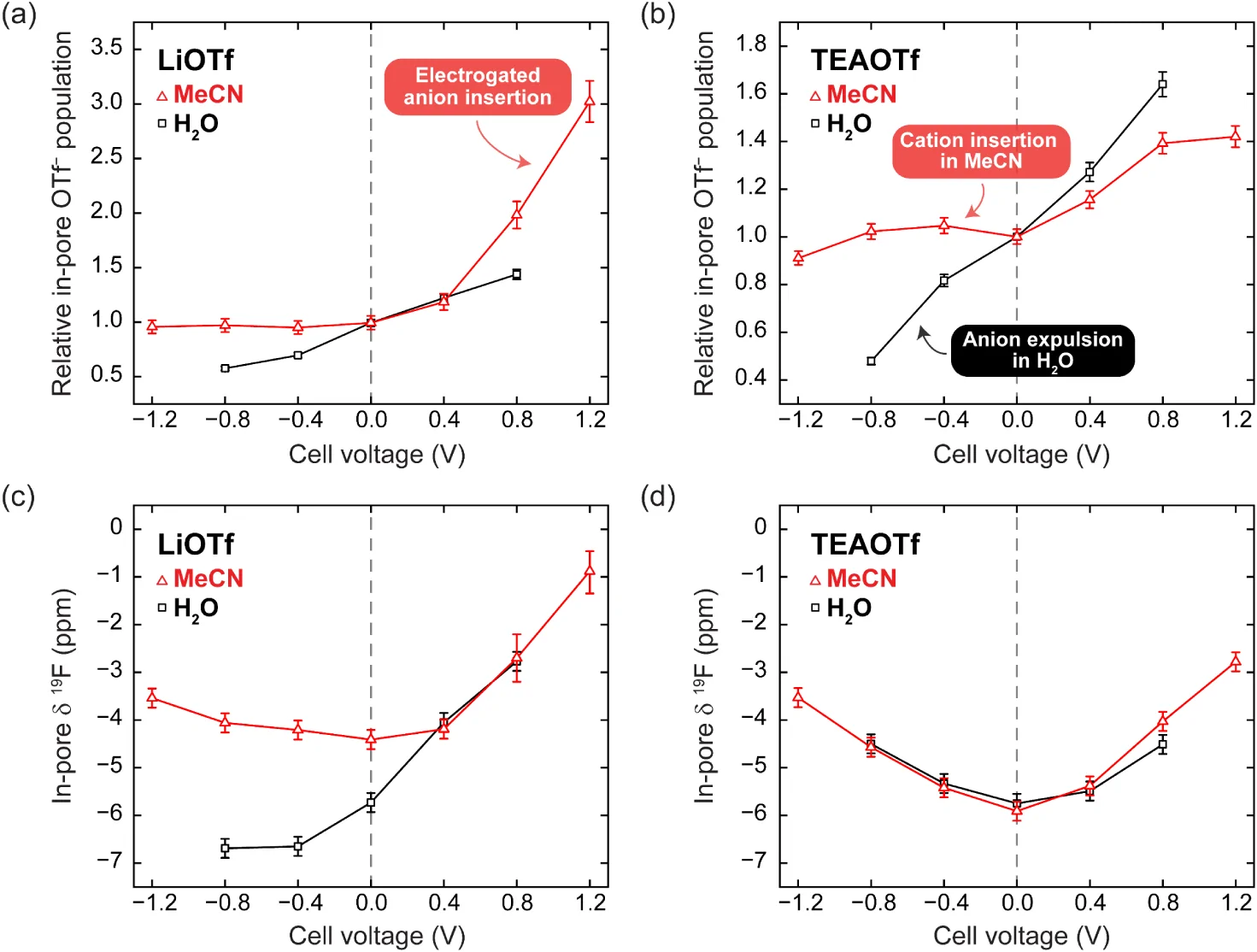
In-pore triflate anion populations against applied cell voltage for *in situ* supercapacitor devices comprising acetonitrile-based (a) LiOTf and (b) TEAOTf. Errors represent an ensemble relative standard deviation across the aqueous systems and MeCN TEAOTf (±3.15%) and for MeCN LiOTf (±6.23%). The increased error for organic LiOTf reflects the constrained model required to stabilize fitting of overlapping resonances. The evolution of the in-pore chemical shift as a function of applied voltage is also shown for (c) LiOTf and (d) TEAOTf. Chemical shift errors (±0.2 ppm) are determined from the full width half maximum (FWHM) of the average in-pore resonance divided by the signal-to-noise ratio. An increased estimated error of ±0.5 ppm is given for the overlapped resonances at +0.8 and +1.2 V for MeCN-based LiOTf.

Under positive electrode polarization, the in-pore OTf^–^ population in MeCN-based LiOTf increases significantly to a relative integral of +3.03 ± 0.19 at a cell voltage of +1.2 V (Figure 7a). We note the spontaneous adsorption of LiOTf in MeCN is very low (Figure 1b); as such, the significant increase in the normalized in-pore OTf^–^ population does not correspond to a large absolute in-pore population. However, the large proportionate increase in the OTf^–^ population reveals a voltage-gated anion insertion mechanism, in which anions that are energetically disfavored from spontaneous pore insertion are readily drawn into the pores when a sufficiently large positive potential is applied.^54^ The potential-driven behavior of the in-pore OTf^–^ population for MeCN-based LiOTf differs substantially from aqueous LiOTf, where a more gradual change in the in-pore OTf^–^ population is observed (Figure 7a).

For TEAOTf, positive electrode polarization increases the in-pore OTf^–^ population for both aqueous and MeCN-based electrolytes, consistent with a counterion adsorption mechanism. Under negative polarization, the in-pore OTf^–^ population for TEAOTf in MeCN remains unchanged across all negative potentials, contrasting the co-ion expulsion mechanism observed for aqueous TEAOTf (Figure 7b). This shows that for TEAOTf in MeCN, OTf^–^ does not partake in the negative charging mechanism, and instead the electrode charge is balanced by the insertion of unobserved TEA^+^ species. Therefore, for 1 M TEAOTf electrolytes, the choice of solvent strongly influences the behavior of OTf^–^ anions in charge-balancing mechanisms within the negative potential range.

Figure 7c,d compare the in-pore ^19^F chemical shift changes measured as a function of cell voltage for MeCN-based and aqueous OTf^–^ electrolytes. For LiOTf in MeCN, the in-pore ^19^F resonance shifts to a less negative chemical shift under negative polarization (Figure 7c). This trend aligns with previous *in situ* ^19^F NMR studies on MeCN-based electrolytes,^16,17,34,53^ but contrasts with the shift toward more negative chemical shifts observed for aqueous LiOTf (Figure 4b). For both aqueous and MeCN-based TEAOTf, the in-pore ^19^F resonance consistently shifts to a less negative chemical shift under positive and negative polarization (Figure 7d). This further suggests that the unique chemical shift behavior is linked to the pronounced anion/cation population imbalances and sizable ΔpH_ex‑pore_ measured for aqueous LiOTf and NaOTf electrolytes and can be at least partially attributed to the presence of H_3_O^+^ in the in-pore environment.

## Conclusions

The NMR data in this work provides new insights into the factors governing spontaneous ion adsorption and the resulting electrochemical properties of aqueous electrolytes. We find that spontaneous adsorption shows a marked dependence on the solvation properties of the individual anions and cations. For electrolyte salts with weakly hydrated ions, such as TEAOTf, both species readily insert into the pores in approximately equal populations under spontaneous adsorption conditions. In contrast, electrolytes with high hydration asymmetry, such as aqueous NaOTf and LiOTf, exhibit a distinct regime. Here, weakly hydrated OTf^–^ anions can readily enter the pores, but their adsorption is limited by electrostatic interactions with strongly hydrated metal cations that are energetically disfavored from pore entry.^24^ In MeCN, the adsorbed ion populations for NaOTf and LiOTf are reduced further relative to aqueous, reflecting increased ion-solvent interaction energies and/or larger effective solvated radii.

For aqueous electrolytes comprising ions with pronounced hydration asymmetry, the resulting in-pore ion imbalances correlate with significant pH increases in the ex-pore electrolyte. This provides compelling evidence for the presence of H_3_O^+^ inside the pores, which compensates for the ionic charge imbalance and allows electrolyte ions to reach equilibrium at an inequivalent distribution reflecting their individual energetic preferences for adsorption. This behavior is further supported by *in situ* NMR measurements, which reveal a decreased OTf^–^ anion involvement for electrolytes with strongly solvated cations, alongside limited changes in the in-pore cation population. These combined observations suggest the participation of compensatory solvent-derived species (H_3_O^+^ and OH^–^) to balance the specific charge.

Regarding electrochemical performance, we establish that a larger disparity between anion and cation hydration properties in aqueous electrolytes is associated with higher specific capacitance. We propose that a proportion of this enhancement arises from the following contributions. First, the localized acidic in-pore environment facilitates surface-redox reactions and provides high-mobility H_3_O^+^ charge carriers within the electric double layer. Second, the initial occupancy of micropores dictates the structural pathway of double-layer formation. In systems with high hydration asymmetry and low spontaneous ion uptake (*e.g*., aqueous NaOTf), the electric double layer is assembled primarily through the progressive adsorption of counterions into relatively vacant pore space. This gradual assembly avoids interfacial congestion inherent to ion-rich pores, where charging necessitates the simultaneous expulsion of co-ions and a redistribution of the existing ionic layer. We suggest this low-concentration ionic state allows for a more energetically optimized ionic configuration, explaining the superior capacitance and improved rate performance observed in electrolytes with the highest hydration asymmetry.

In MeCN-based electrolytes, where H_3_O^+^ and OH^–^ populations are negligible, the link between solvation properties and capacitive performance is governed by strict stoichiometry. MeCN-based electrolytes exhibit lower spontaneous adsorption and lower specific capacitance, with a performance trend that is reversed relative to aqueous systems. This suggests that in the absence of compensatory water species, capacitance is dominated by steric factors, such as ion/pore size matching and the energetic cost of desolvation. For MeCN-based LiOTf, the rapid increase in ion concentration during charging signifies a voltage-triggered “ion-gating” phenomenon.

Overall, these results establish a molecular-level framework for controlling electrosorption and capacitance through the rational selection of ion-solvent pairs. By leveraging hydration asymmetry as a design principle, the in-pore pH and specific capacitance can be precisely tuned. This work highlights the fundamental role of ion-solvent interactions in developing next-generation electrolytes for specific electrochemical use cases, from high-power supercapacitors to water desalination.

## Supplementary Material



## Data Availability

All data accompanying this publication is freely available at https://doi.org/10.17635/lancaster/researchdata/746.

## Notes

Table: CHNS-O elemental analysis of ACC-5092-20; MAS NMR spectra of 1 M aqueous TEACl compared to 1 M aqueous NaCl; changes in H_3_O^+^ population calculated from pH changes; in-pore ion population normalized capacitance; rate performance data; charge data for *in situ* NMR measurements; analysis of in-pore environment chemical shifts with respect to applied voltage for a range of aqueous salts (PDF)
