# Supplementary material for: Hydration Asymmetry and Solvent-Mediated Charge Balancing in Microporous Carbons: An NMR Study of Aqueous and Organic Electrosorption
Source: J Am Chem Soc. 2026 Sep 12;148(44):47785–97. doi: 10.1021/jacs.6c08967 (PMC13761276; doi:10.1021/jacs.6c08967)
Supplement: Supplementary file 1 [file ja6c08967_si_001.pdf]

Supporting Information

Hydration Asymmetry and Solvent-Mediated Charge Balancing in Microporous Carbons: An NMR Study of Aqueous and Organic Electrosorption

Ryan J. Bragg[1,2], Kieran Griffiths[1], Chris W. Cook[1,2], Robert A. W. Dryfe[3,4], Volker Presser[5,6,7], and John M. Griffin[1*]

[1] Department of Chemistry, Lancaster University, Lancaster, LA1 4YB, United Kingdom
[2] Department of Chemistry, University of Warwick, Coventry, CV4 7AL, United Kingdom
[3] Department of Chemistry, University of Manchester, Oxford Road, Manchester, M13 9PL, United Kingdom
[4] Henry Royce Institute, University of Manchester, Oxford Road, Manchester, M13 9PL, United Kingdom
[5] INM - Leibniz Institute for New Materials, Campus D2 2, 66123, Saarbrücken, Germany
[6] Department of Materials Science & Engineering, Saarland University, Campus D2 2, 66123, Saarbrücken, Germany
[7] saarene - Saarland Center for Energy Materials and Sustainability, Campus C4 2, 66123, Saarbrücken, Germany

* Corresponding author. E-mail address: j.griffin@lancaster.ac.uk

Tables

Table S1 – (a) Element composition of activated carbon cloth ACC-5092-20. The elements H, C, N were analyzed by using an Elementar vario Micro Cube for CHNS analysis and an Elementar rapid OXY cube was used for O determination. Both N and S were below the limit of detection.

Element	Mass percentage (%)
**Carbon**	97.02±0.64
**Hydrogen**	0.570±0.041
**Oxygen**	1.108±0.068
**Nitrogen**	N/a
**Sulphur**	N/a

Figures

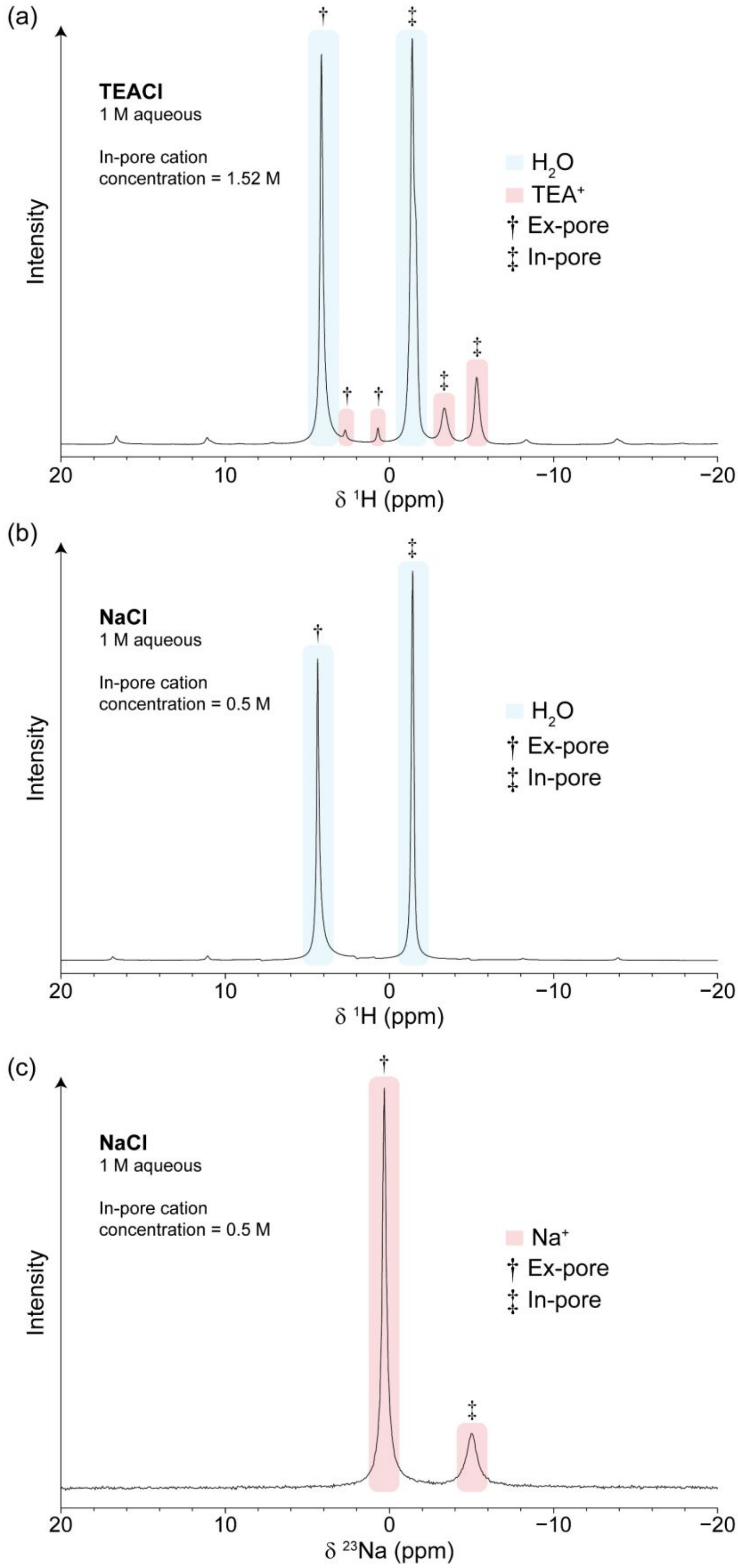

Figure S1 – (a) ^{1}H MAS NMR spectrum of ACC-20 (13 mg) loaded with 1 M aqueous TEACl (15 µL). (b) ^{1}H and (c) ^{23}Na MAS NMR spectrum of ACC-20 (13 mg) loaded with 1 M aqueous NaCl (15 µL). Cations and H_2O are highlighted by red and blue boxes, respectively. TEACl exhibits a larger in-pore cation resonance relative to its ex-pore cation resonance, whereas NaCl exhibits the inverse.

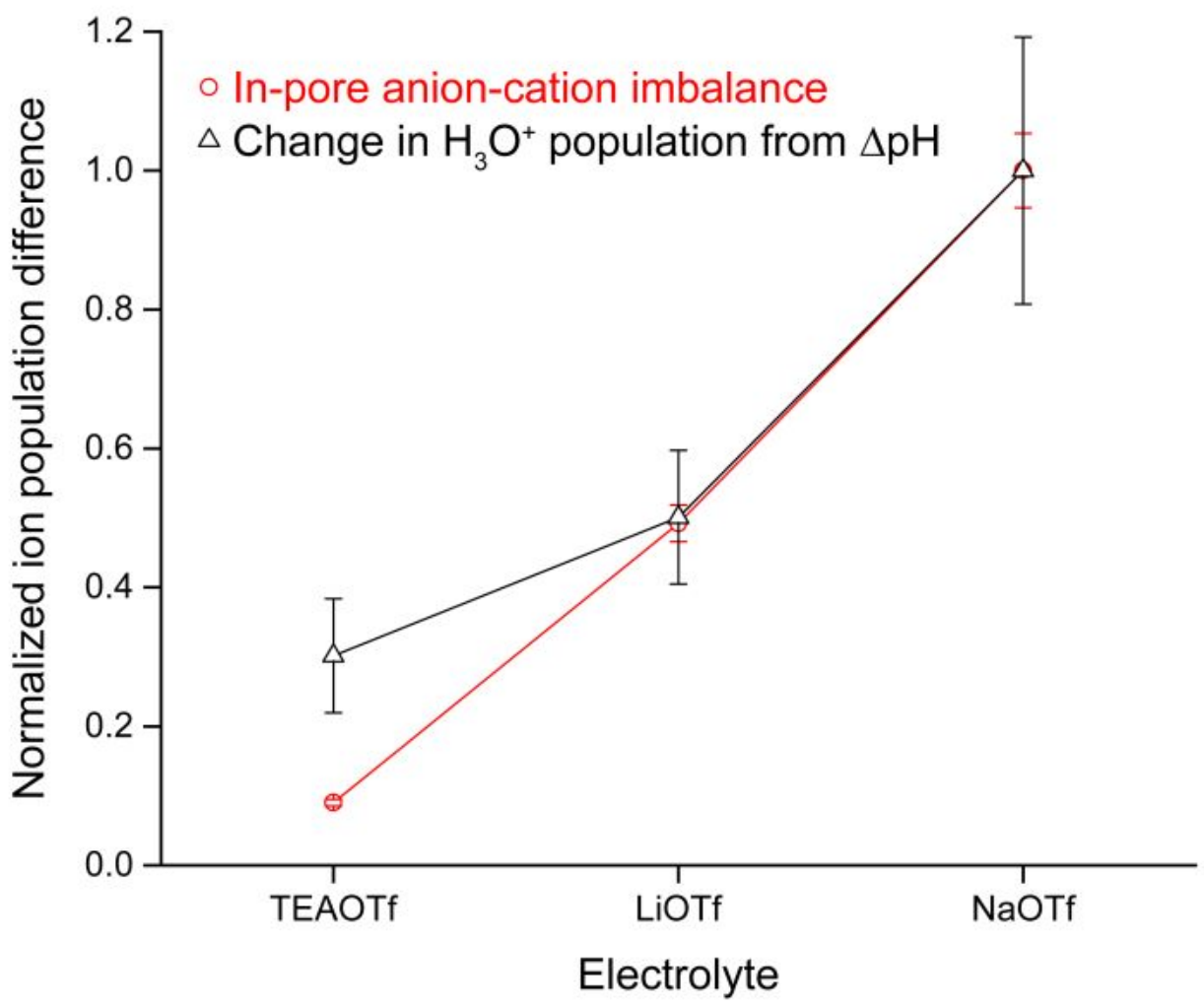

Figure S2 – Relative change in H_3O^+ population upon addition of ACC-20 (calculated from ΔpH) compared with the relative in-pore anion-cation population imbalance determined from MAS NMR experiments on aqueous triflate electrolytes (13 mg ACC-20, 15 µL). All values are normalized relative to the respective values obtained for NaOTf. Error bars for NMR data represent the relative standard deviation across 3 independent spectral deconvolution fits (±2.5%). Error bars for OH^- population changes represent propagated uncertainties derived from the pooled standard deviation of 3 independent pH measurements (±0.06 pH units).

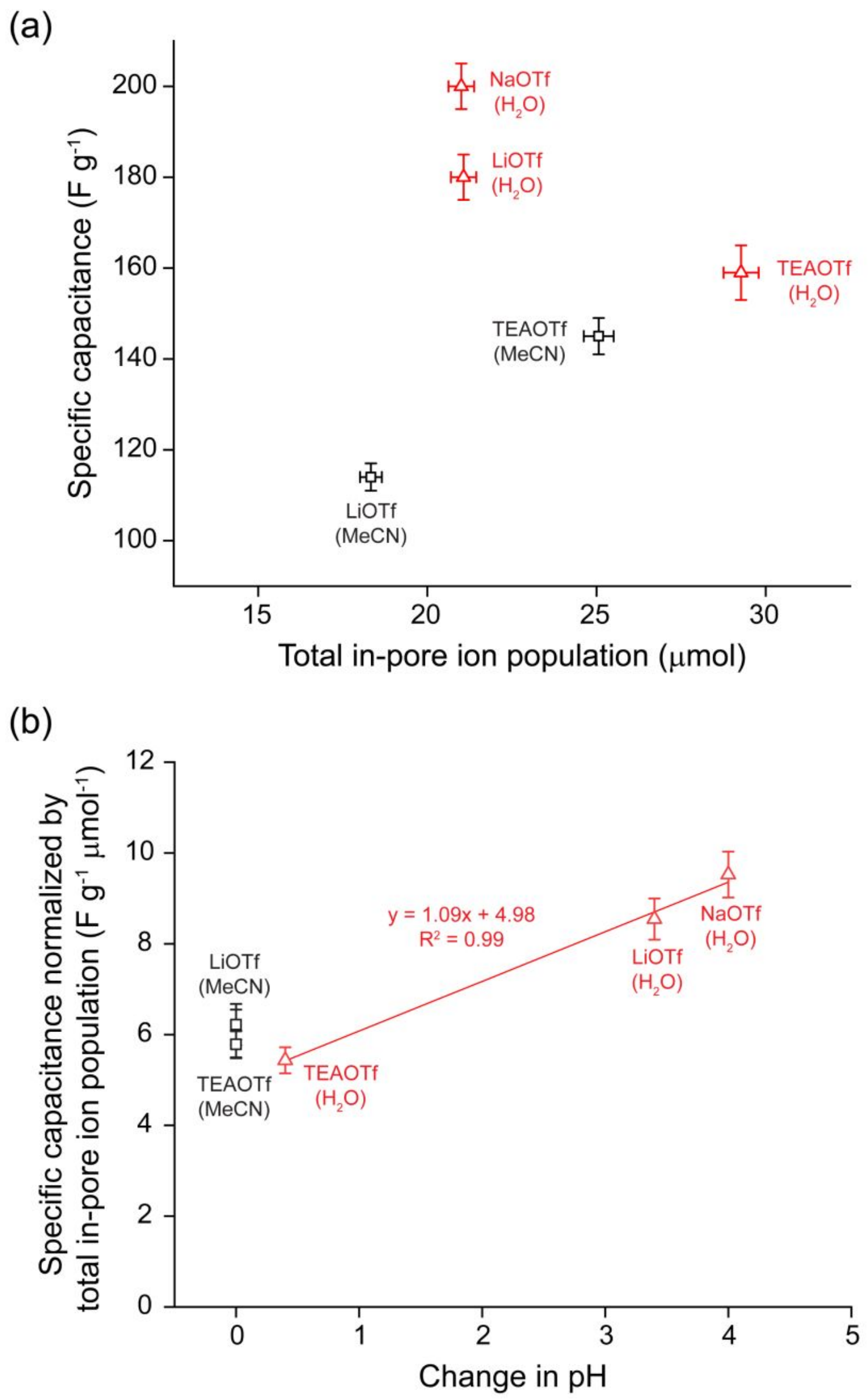

Figure S3 – (a) Specific capacitance values measured from full cyclic voltammograms plot against the total (summed anion and cation) in-pore ion population, as measured by solid-state NMR under MAS at 0 V, for ACC-20 (13 mg) loaded with 1 M electrolyte (15 µL) (b) Specific capacitance values normalized by the total in-pore ion population versus the measured pH change of the electrolyte upon the addition of ACC-20. Aqueous electrolytes are shown in red, and MeCN-based electrolytes are displayed in black. We note that pH for MeCN-based electrolytes is not measurable; these points are included on the plot at an arbitrary pH = 0 for comparison with the datapoints for aqueous electrolytes. For aqueous electrolytes, TEAOTf shows a comparable capacitance to MeCN-based electrolytes, while an increased capacitance per ion is observed for electrolytes in which a sizeable pH change is observed.

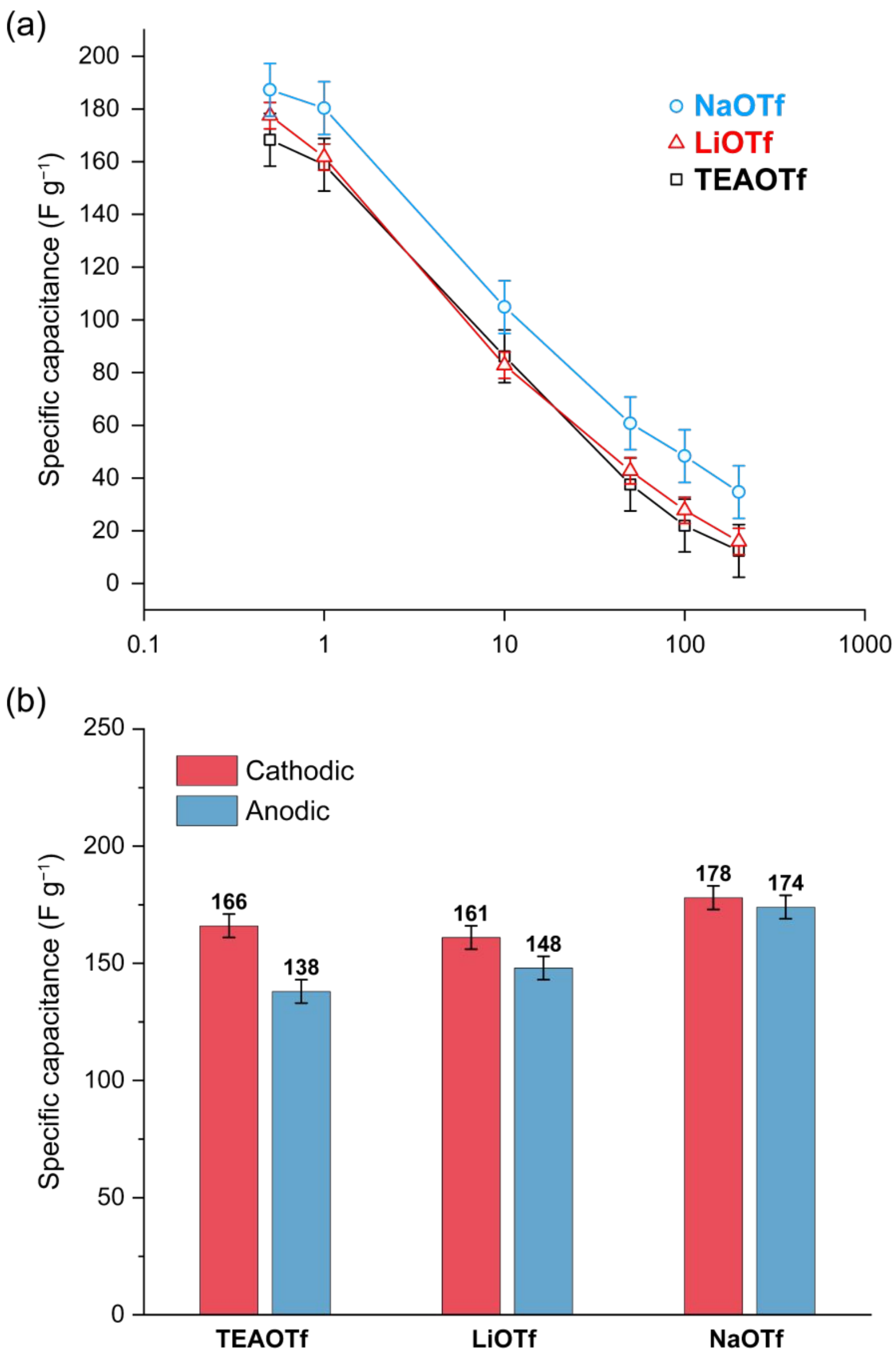

Figure S4 – (a) Rate performance data (specific capacitance vs. scan rate) derived from three-electrode half-cell cyclic voltammetry experiments (0 V to +0.8 V *vs.* Ag/AgCl) for 1 M aqueous NaOTf, LiOTf, and TEAOTf. (b) Comparison of specific capacitances derived from three-electrode cyclic voltammetry experiments across positive (0 V to +0.8 V) and negative (0 V to -0.8 V) potential windows. Error bars for specific capacitance are derived from the propagation of instrumental weighing uncertainty ±0.1 mg relative to the electrode mass.

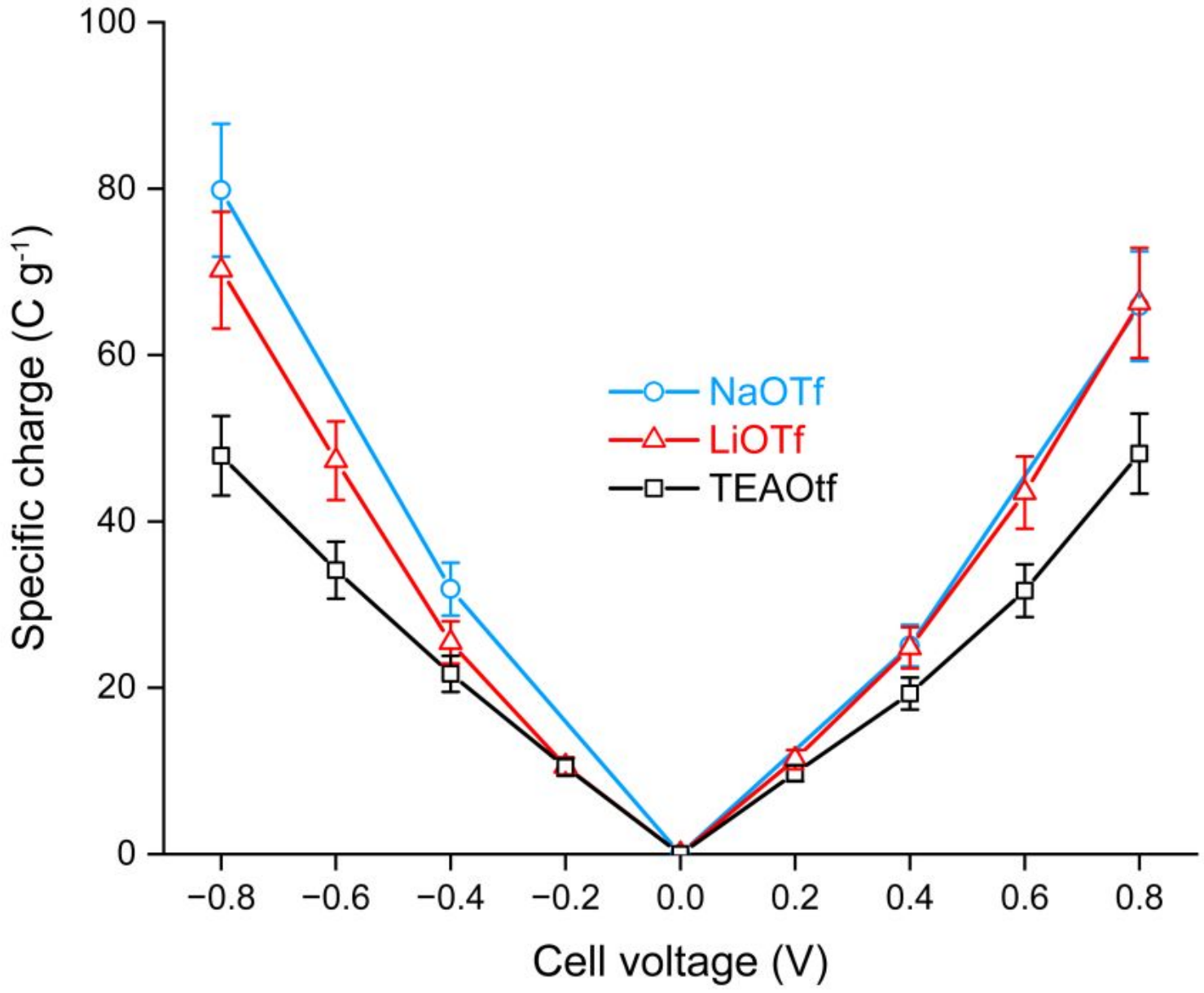

Figure S5 – Specific charge with respect to the applied cell voltage obtained during the step-wise voltage holds applied during *in situ* measurements of the aqueous electrolytes LiOTf, NaOTf, and TEAOTf.

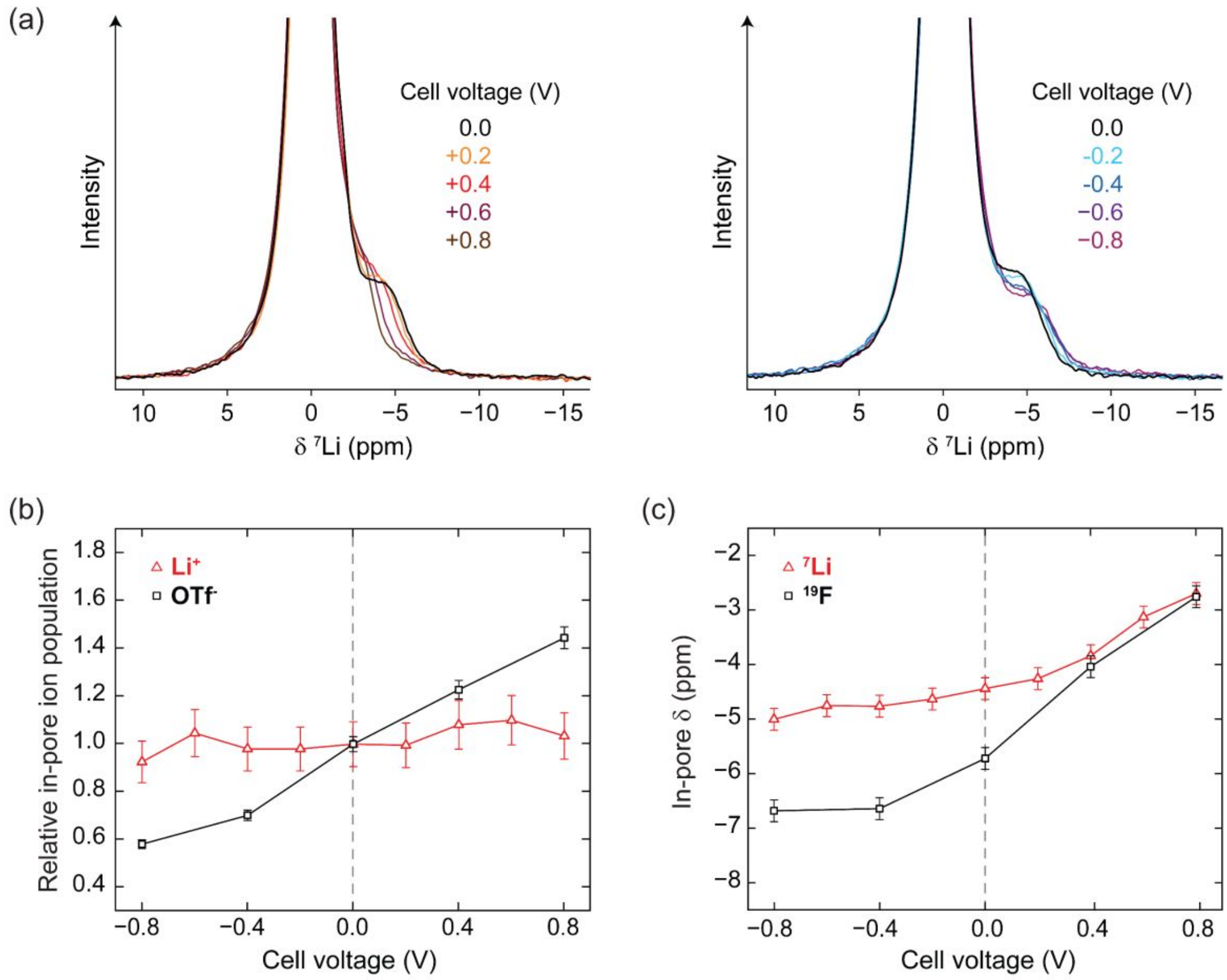

Figure S6 – (a) *In situ* ^{7}Li NMR spectra of 1 M aqueous LiOTf, acquired across different applied potentials. Spectra are referenced such that the center of the ex-pore triflate resonance is at 0 ppm. (b) Voltage-dependent response of in-pore lithium cation and triflate anion populations. Error bars represent an ensemble relative standard deviation of 9.4% and 3.15%, respectively, each derived from 3 independent fits. (c) ^{7}Li and ^{19}F chemical shifts versus cell voltage for in-pore Li^{+} and OTf^{-}, respectively. Chemical shift errors (±0.2 ppm) are determined from the full width half maximum (FWHM) of the average in-pore resonance divided by the signal-to-noise ratio. Note that ^{7}Li and ^{19}F data were obtained on separate cells.

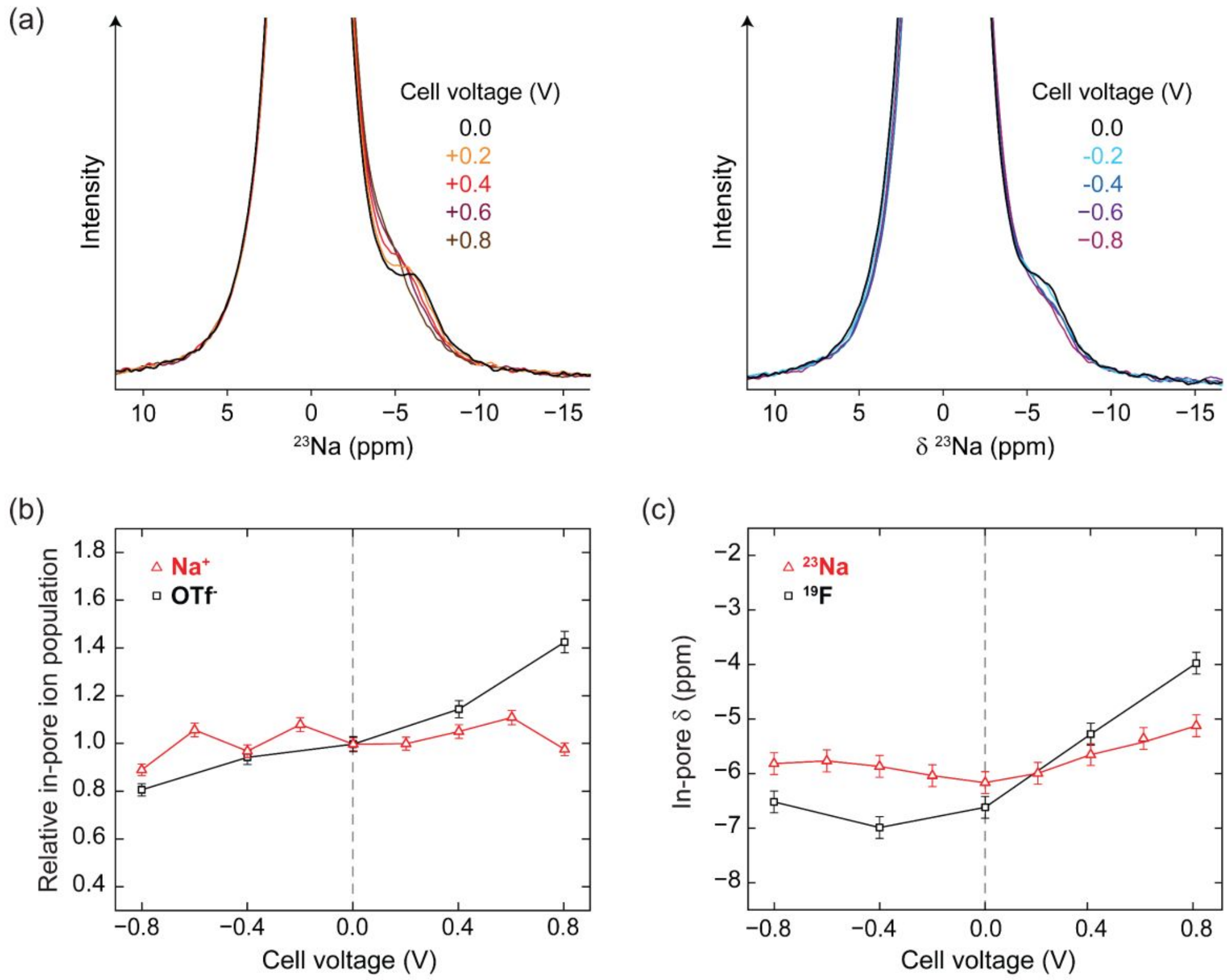

Figure S7 – (a) *In situ* ^{23}Na NMR spectra of 1 M aqueous NaOTf, acquired across different applied potentials. Spectra are referenced such that the center of the ex-pore triflate resonance is at 0 ppm. (b) Voltage-dependent response of in-pore lithium cation and triflate anion populations. Error bars represent an ensemble relative standard deviation of 2.7% and 3.15%, respectively, each derived from 3 independent fits. (c) ^{23}Na and ^{19}F chemical shifts versus cell voltage for in-pore Na^+ and OTf^-, respectively. Chemical shift errors (±0.2 ppm) are determined from the full width half maximum (FWHM) of the average in-pore resonance divided by the signal-to-noise ratio. Note that ^{23}Na and ^{19}F data were obtained on separate cells.

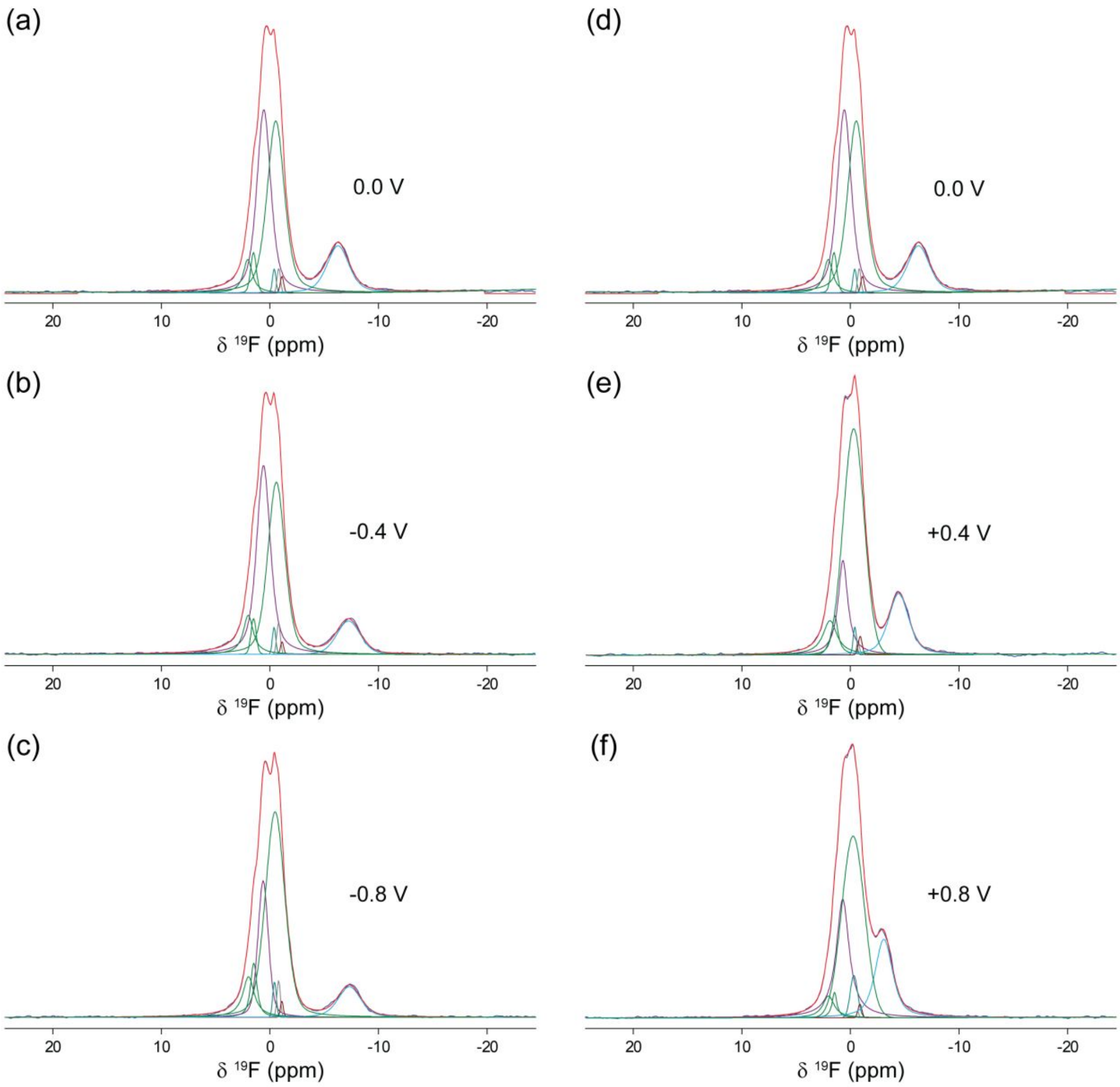

Figure S8 – Fitted deconvolution of *in situ* ^{19}F NMR spectra measured for aqueous 1 M LiOTf in ACC-20 across (a-c) negative and (d-f) positive electrode potentials. In-pore resonance is fitted by the light-blue peak. Minor sharp resonances between 3 ppm and 0 ppm are used to fit sharp components arising from bulk magnetic susceptibility effects arising from metallic components within the cell.

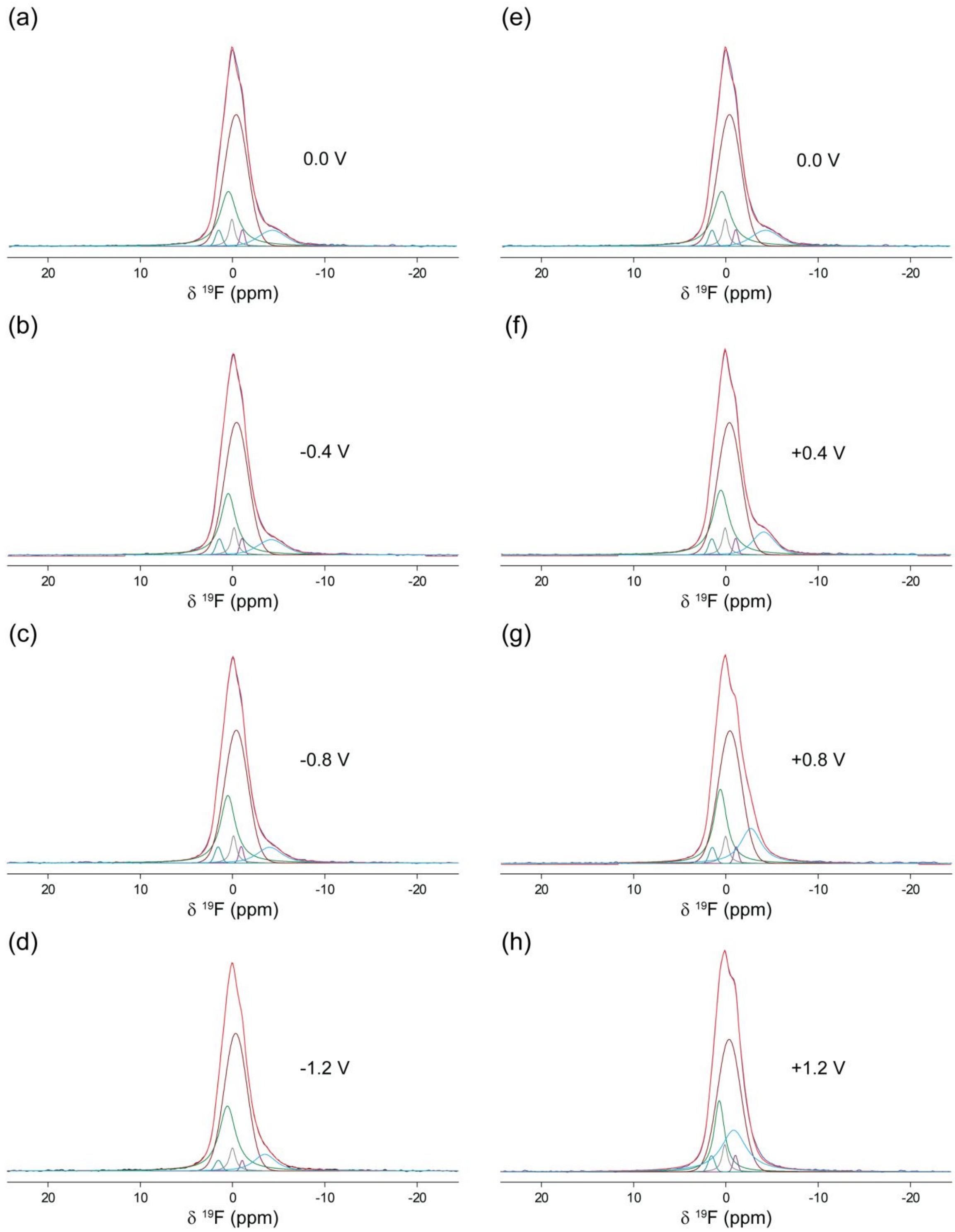

Figure S9 – Fitted deconvolution of *in situ* ^{19}F NMR spectra measured for MeCN-based 1 M LiOTf in ACC-20 across (a-d) negative and (e-h) positive electrode potentials. In-pore resonance is fitted by the light-blue peak. Significant overlap of the ex-pore and in-pore resonances in this system required the major ex-pore component (purple fitted peak) to be fixed in intensity and width to stabilize other fitted parameters. Minor sharp resonances between 3 ppm and 0 ppm are used to fit sharp components arising from bulk magnetic susceptibility effects arising from metallic components within the cell.

Deconvolution methodology

Fitting of the *in situ* NMR spectra are challenging owing to bulk magnetic susceptibility contributions from the carbon cloth itself alongside the metallic components required for cycling. *In situ* NMR spectra were first reprocessed to remove the exponential multiplication window function from the time domain data (line broadening) to identify sharp components in the ex-pore resonance corresponding to bulk magnetic susceptibility effects on the electrolyte. Small (<2 % of total integral) peaks were then positioned at the chemical shifts of these sharp features to capture their influence on the ex-pore lineshape. Line broadening (100 Hz) was then reapplied to better identify lineshapes from the noise.

Two larger peaks were then used to fit the overall shape of the ex-pore resonance with a third and final peak being used to fit the in-pore resonance. For the fitting of the aqueous electrolytes and MeCN-based TEAOTf, the peak amplitude, width, position, and Gaussian-to-Lorentzian lineshape ratio of each peak was allowed to vary freely to give the best fit with experimental. For MeCN-based LiOTf, the minor peaks from susceptibility and one of the peaks for the ex-pore were fixed to reproducibly fit the spectra. The fitting was first performed on the 0 V system in each instance, with the resulting best fit being applied to the next *in situ* spectrum in the potential step series (*e.g.*, 0 V to +0.2 V) and the fit was then reoptimized. Explicit care has been taken here to ensure that the parameters of the ex-pore fits remain consistent across the different spectral fits for each system (LiOTf, NaOTf, TEAOTf) to accurately map the changes explicitly related to the in-pore resonance. Once each of the *in situ* spectra for each electrolyte were fitted, the fits of the spectra corresponding to the most extreme potentials (*e.g.,* +0.8 V and -0.8 V for aqueous systems, and +1.2 V and -1.2 V for organic systems) were directly compared to ensure a relative consistency of the components comprising the ex-pore fit as to minimize significant changes in these fits dominating changes observed in the in-pore resonance.

The fitted in-pore resonances were then integrated to determine the total spin population of that site. These integrals were then normalized against the 0 V integral to determine the relative change of in-pore population with respect to the electrode potential. To quantify the fitting uncertainty, the entire deconvolution approach was then repeated for each spectrum with a reduced line broadening (50 Hz). The integral of the in-pore resonance with 50 Hz and 100 Hz of line broadening were compared, with the relative standard deviation (RSD) being calculated for each point. This was then repeated a final time for each spectra following a fresh Fourier transform of the time-domain data and a new rephasing of the spectra using a line broadening of 100 Hz. The integral of the in-pore resonance between the original and rephased data was compared, with the RSD error being calculated for each point.

The variance from both line broadening and rephasing were pooled via root mean square deviation (RMSD) propagation to determine the relative population error for each electrolyte. For systems exhibiting comparable in-pore resolution (aqueous LiOTf, NaOTf, and TEAOTf, and MeCN-based TEAOTf), the ensemble error was calculated by pooling these variances across all potentials, yielding a final ensemble error of ±3.15%. For MeCN-based LiOTf, the error was not pooled with the other electrolytes due to significant overlap between the in-pore and ex-pore necessitating fixing of fitting parameters. The resultant error for this system is notably elevated (±6.23%).

Uncertainties in the chemical shift positions are fundamentally determined by the spectral resolution limits of the *in situ* measurements rather than by fitting variance. The positional error was estimated as the average full width half maximum (FWHM) divided by the signal-to-noise ratio (SNR) of the in-pore resonance, yielding a baseline uncertainty of ±2.0 ppm. For the overlapped MeCN-based LiOTf resonances at +0.8 V and +1.2 V, this estimated positional error is increased to ±0.5 ppm to account for the reduced discrete resolution of the overlapping components.
